# Six1 proteins with human branchio-oto-renal mutations differentially affect cranial gene expression and otic development

**DOI:** 10.1242/dmm.043489

**Published:** 2020-03-03

**Authors:** Ankita M. Shah, Patrick Krohn, Aparna B. Baxi, Andre L. P. Tavares, Charles H. Sullivan, Yeshwant R. Chillakuru, Himani D. Majumdar, Karen M. Neilson, Sally A. Moody

**Affiliations:** 1 Department of Anatomy and Cell Biology, George Washington University School of Medicine and Health Sciences, Washington, DC 20037, USA; 2 Institute of Zoology, University of Hohenheim, Stuttgart 70599, Germany; 3 Department of Biology, Grinnell College, Grinnell, IA 50112, USA

**Keywords:** Neural crest, Preplacodal ectoderm, Cranial placodes, Eya1, Otolith, Otocyst

## Abstract

Single-nucleotide mutations in human *SIX1* result in amino acid substitutions in either the protein-protein interaction domain or the homeodomain, and cause ∼4% of branchio-otic (BOS) and branchio-oto-renal (BOR) cases. The phenotypic variation between patients with the same mutation, even within affected members of the same family, make it difficult to functionally distinguish between the different *SIX1* mutations. We made four of the BOS/BOR substitutions in the *Xenopus* Six1 protein (V17E, R110W, W122R, Y129C), which is 100% identical to human in both the protein-protein interaction domain and the homeodomain, and expressed them in embryos to determine whether they cause differential changes in early craniofacial gene expression, otic gene expression or otic morphology. We confirmed that, similar to the human mutants, all four mutant *Xenopus* Six1 proteins access the nucleus but are transcriptionally deficient. Analysis of craniofacial gene expression showed that each mutant causes specific, often different and highly variable disruptions in the size of the domains of neural border zone, neural crest and pre-placodal ectoderm genes. Each mutant also had differential effects on genes that pattern the otic vesicle. Assessment of the tadpole inner ear demonstrated that while the auditory and vestibular structures formed, the volume of the otic cartilaginous capsule, otoliths, lumen and a subset of the hair cell-containing sensory patches were reduced. This detailed description of the effects of BOS/BOR-associated *SIX1* mutations in the embryo indicates that each causes subtle changes in gene expression in the embryonic ectoderm and otocyst, leading to inner ear morphological anomalies.

## Abstract (precis)

**Summary:** Branchio-otic/branchio-oto-renal syndromes result in craniofacial defects including deafness. Four of the known human *SIX1* mutations cause differential changes in craniofacial gene expression and otic morphology when expressed in *Xenopus* embryos.

## INTRODUCTION

Branchio-otic (BOS) and branchio-oto-renal (BOR) syndromes together comprise the second most prevalent birth defect involving hearing loss (Hilgert et al., 2009; Smith, 2018). These syndromes, which are inherited in an autosomal dominant manner with 100% penetrance (Chang et al., 2004; Fraser et al., 1980), are characterized by deformities of the external, middle and inner ear structures, as well as second branchial arch defects that include fistulas and cysts; renal malformations also characterize BOR (Kochhar et al., 2007; Smith, 2018). External ear malformations include pre-auricular pits, tags and cupped/lop ears; those of the middle ear include ossicle deformities, and of the inner ear include auditory and vestibular canal hypoplasia (Smith, 2018). There is considerable variability in the severity, presence and type of these abnormalities among patients, including family members carrying the same mutation. How these varied defects arise in the embryo has not yet been addressed.

**Fig. 1.**
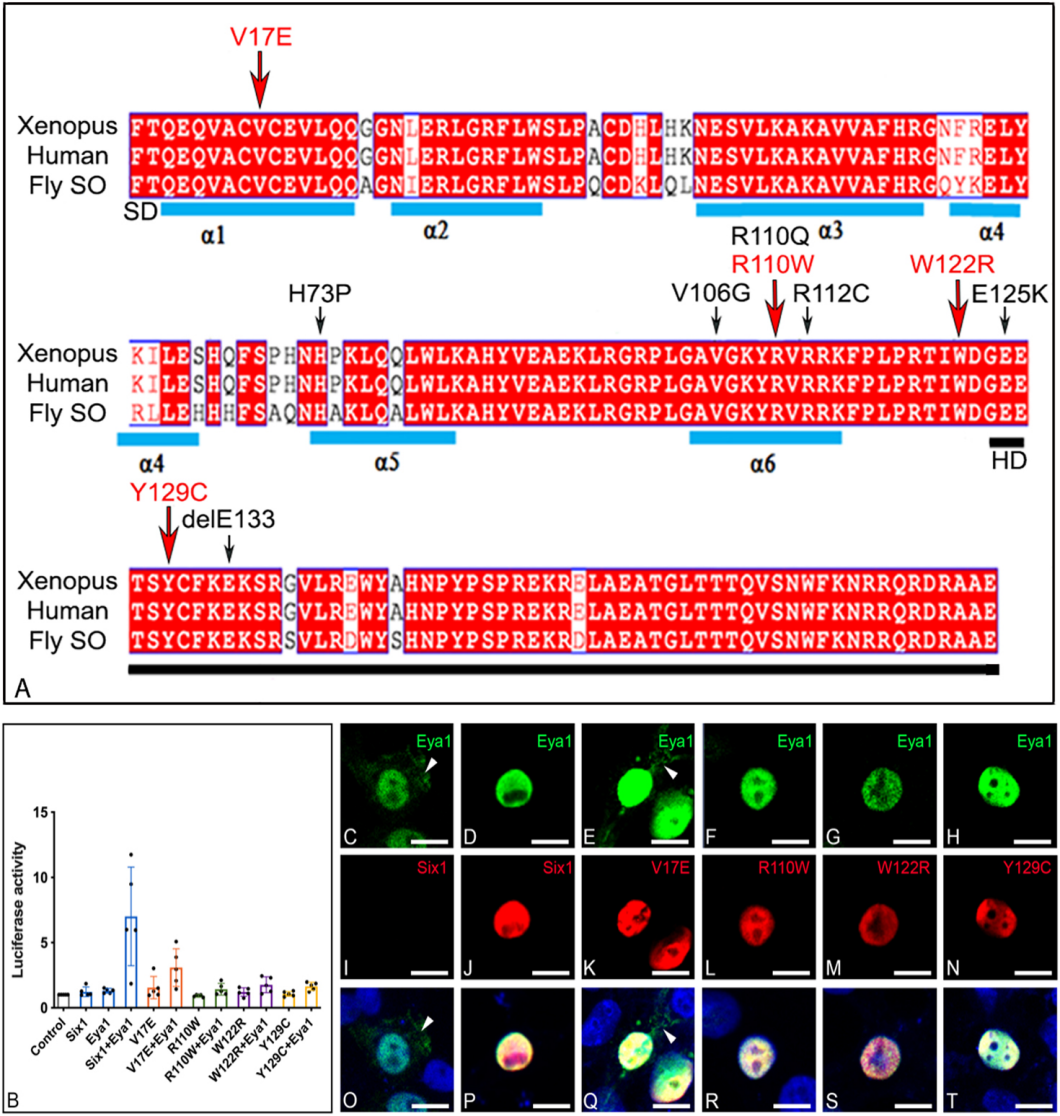
**BOS/BOR mutations and their transcriptional effects.** (A) Amino acid alignment of the N-terminal region of *Xenopus laevis* Six1, human SIX1 and *Drosophila* Sine oculis (SO) shows a high level of identity across species; human and frog are 100% identical in this region; differences from fly are in white. The sequence shown begins with the six domain (SD), which contains six α-helices (blue bars), and ends with the homeodomain (HD, black bar). Amino acid substitutions/deletions that have been reported in human BOS/BOR patients are indicated with arrows; red arrows indicate the four mutations that were examined in this study. (B) Expression of Six1+Eya1 caused a significant ∼7-fold increase in luciferase activity when compared to activity of control vector (*P*<0.0001), Six1WT alone (*P*<0.0001) or Eya1 alone (*P*<0.0001). Each mutant plus Eya1 failed to significantly induce luciferase activity relative to control (V17E, *P*=0.27122; R110W, *P*=0.99999; W122R, *P*=0.99764; Y129C, *P*=0.99947) or in the absence of Eya1 (V17E, *P*=0.99988; R110W, *P*=0.99999; W122R, *P*=0.99999; Y129C, *P*=0.99999). Experiments were repeated five independent times and subjected to a one-way ANOVA with Tukey post hoc multiple comparisons test. Bars=mean±s.d. (C,I,O). HEK293T cells transfected with only Myc-Eya1 show both cytoplasmic (arrowheads) and nuclear localization. (D,J,P) Cells co-transfected with both Six1WT-FLAG and Myc-Eya1 show nuclear colocalization of both proteins. (E,K,Q) Those transfected with both V17E-FLAG and Myc-Eya1 showed nuclear colocalization and some cytoplasmic Eya1 (arrowheads). Those transfected with (F,L,R) R110W-FLAG and Myc-Eya1, (G,M,S) W122R-FLAG and Myc-Eya1, or (H,N,T) Y129C-FLAG and Myc-Eya1 showed exclusive nuclear colocalization. Scale bars: 10 µm.

Mutations in two genes have been identified in about half of patients with BOS/BOR (reviewed in Smith, 2018; Moody et al., 2015). The gene encoding the SIX1 transcription factor is mutated in ∼4% of patients (BOS3/BOR3), and the gene encoding EYA1, which binds to SIX1 to modify its transcriptional activity, is mutated in ∼40% of the patients (BOS1/BOR1). SIX1 is highly related to *Drosophila* Sine oculis (SO) (Cheyette et al., 1994; Serikaku and O'Tousa, 1994; Kawakami et al., 1996). Like other proteins of the SIX family, SIX1 possesses two highly conserved domains: a SIX-type homeodomain (HD) that binds to DNA and an N-terminal SIX domain (SD) that binds to cofactor proteins including EYA1 (Pignoni et al., 1997; Kawakami et al., 2000; Kobayashi et al., 2001; Kenyon et al., 2005a). Several *SIX1* single-nucleotide mutations that result mostly in amino acid substitutions have been reported in BOS/BOR patients (Fig. 1A), but only a few have been functionally analyzed and only in cell culture. The V17E mutation in the first α-helix of the SD is reported to abolish the SIX1-EYA interaction and subsequent translocation of EYA to the nucleus (Patrick et al., 2009). The R110W mutation in the sixth α-helix of the SD is reported to decrease the SIX1-EYA interaction (Kochhar et al., 2008; Ruf et al., 2004) and be deficient in transcriptional activation (Patrick et al., 2009). The W122R mutation, adjacent to the HD (Sanggaard et al., 2007), is predicted to affect DNA binding (Patrick et al., 2009), and the Y129C mutation in the HD results in significantly reduced DNA binding (Ruf et al., 2004; Patrick et al., 2009). To our knowledge, the gene expression consequences of these mutations on craniofacial development are unknown.

The craniofacial structures affected in BOS/BOR are derived from non-neural ectoderm (external ear epidermis, external auditory canal), cranial neural crest (second branchial arch, middle ear ossicles, external ear cartilage) and otic placode [inner ear, auditory-vestibular ganglion (VIIIg)] (Groves and Fekete, 2012; Moody et al., 2015). The otic placode is specified at neural plate stages, invaginates from the surface ectoderm to form a flattened otic vesicle, and then progresses through complex morphogenesis to form an inner ear comprised of both ventral auditory and dorsal vestibular subdomains (Alsina and Whitfield, 2017). In general, the morphological changes that transform the otic vesicle from an epithelial sac into an elaborate fluid-filled labyrinth are conserved across vertebrates (Bever et al., 2003; Barald and Kelley, 2004; Quick and Serrano, 2005; Groves and Fekete, 2012; Alsina and Whitfield, 2017). Studies in several vertebrates indicate that *Six1* is a crucial regulator of cranial placode development (Brugmann and Moody, 2005; Grocott et al., 2012; Moody and LaMantia, 2015; Moody and Saint-Jeannet, 2014; Saint-Jeannet and Moody, 2014; Schlosser, 2006, 2010; Streit, 2007). Six1 loss-of-function studies demonstrate reduced expression of several placode genes and defects in otic development, including loss of hair cells and defective patterning of the sensory epithelium in which they reside (Laclef et al., 2003; Zheng et al., 2003; Brugmann et al., 2004; Ozaki et al., 2004; Schlosser and Ahrens, 2004; Zou et al., 2004; Bricaud and Collazo, 2006; Konishi et al., 2006; Ikeda et al., 2007; Chen et al., 2009; Christophorou et al., 2009; Ikeda et al., 2010; Bricaud and Collazo, 2011; Zhang et al., 2017; Sullivan et al., 2019). *Six1* gain of function expands placode domains at the expense of the adjacent epidermal and neural crest regions (Brugmann et al., 2004; Schlosser et al., 2008; Christophorou et al., 2009; Maharana and Schlosser, 2018), and promotes utricular hair cell over VIIIg neuron formation (Bricaud and Collazo, 2006).

Given the key role of *Six1* during early development of the inner ear and that known human mutations in this gene result in BOS/BOR, our goal was to determine whether these mutations have differential effects on the development of the cranial neural crest and placodes, the precursor populations that contribute extensively to the dysmorphic tissues. Using *Xenopus*, which evolved for terrestrial hearing and therefore extensively shares otic development, morphology and gene expression with mammals, we expressed *Xenopus* Six1 proteins carrying the human mutations in a wild-type (WT) background and assessed their effects on early gene expression and otic morphology. Each mutant caused specific and often different disruptions in the size of the domains of several neural border, neural crest and pre-placodal ectoderm genes. Each mutant also had differential effects on otic vesicle genes and tadpole inner ear morphology. This detailed description of early gene expression changes caused by *SIX1* mutations suggests that subtle changes in their patterns in the embryonic ectoderm and otocyst contribute to the variable BOS/BOR patient phenotypes.

## RESULTS

### *Xenopus Six1* mutants carrying human mutations do not activate Eya1-mediated transcription

Previous studies in human cell lines demonstrated that V17E, R110W and Y129C mutations render SIX1 unable to activate transcription in the presence of mouse Eya1 or human EYA2 (Ruf et al., 2004; Patrick et al., 2009). We observed the same with the *Xenopus* mutants that we generated (Fig. 1B). Co-transfection of HEK293T cells with WT *Xenopus Six1* (*Six1WT*) plus *Eya1* caused a ∼7-fold increase (*P*<0.0001) in luciferase activity relative to levels after transfection of control vector, *Six1WT* alone or *Eya1* alone. All four *Xenopus Six1* mutants failed to significantly induce luciferase activity over control levels either alone (V17E, *P*=0.99988; R110W, *P*=0.99999; W122R, *P*=0.99999; Y129C, *P*=0.99999) or in the presence of Eya1 (V17E, *P*=0.27122; R110W, *P*=0.99999; W122R, *P*=0.99764; Y129C, *P*=0.99947). To exclude the possibility that the lack of transcriptional activation was caused by the mutations blocking Six1 from either localizing to the cell nucleus or translocating Eya1 to the nucleus, cells were co-transfected with FLAG-tagged versions of *Six1WT* or each *Six1* mutant plus Myc-tagged Eya1 followed by confocal immunofluorescence microscopy (Fig. 1C-T). Consistent with the biochemical results of Patrick et al. (2009), we observed that Six1WT and all four Six1 mutants were located almost exclusively in the nucleus. Thus, the nuclear localization of *Xenopus* Six1 is not disrupted by these mutations, even though the nuclear localization signal is likely to be located in the SD (Brodbeck et al., 2004). This assay also showed that R110W, W122R and Y129C efficiently translocate all of the transfected Eya1 into the nucleus; in no cell was Eya1 detected in the cytoplasm. However, whereas Patrick et al. (2009) showed that V17E is unable to translocate EYA2 to the nucleus, we observed Eya1 immunofluorescence in the cytoplasm and the nucleus (Fig. 1E,Q). This discrepancy is likely because instead of the mammary cancer cells used by Patrick et al. (2009), we used HEK293T cells, which are known to express low levels of SIX1 (see Materials and Methods); thus, some Eya1 is translocated to the nucleus even in the absence of Six1 transfection. In agreement with Patrick et al. (2009), we found V17E to be the only mutant that had impaired transport of Eya1 into the nucleus. Overall, these results demonstrate that the *Xenopus* constructs we created to model the BOS/BOR mutations are translated, and even though they access the nucleus, they are transcriptionally deficient in the presence of *Xenopus* Eya1. These results match the biochemical analyses of the mouse and human mutated proteins.

### Six1 mutants alter craniofacial gene expression

**Fig. 2.**
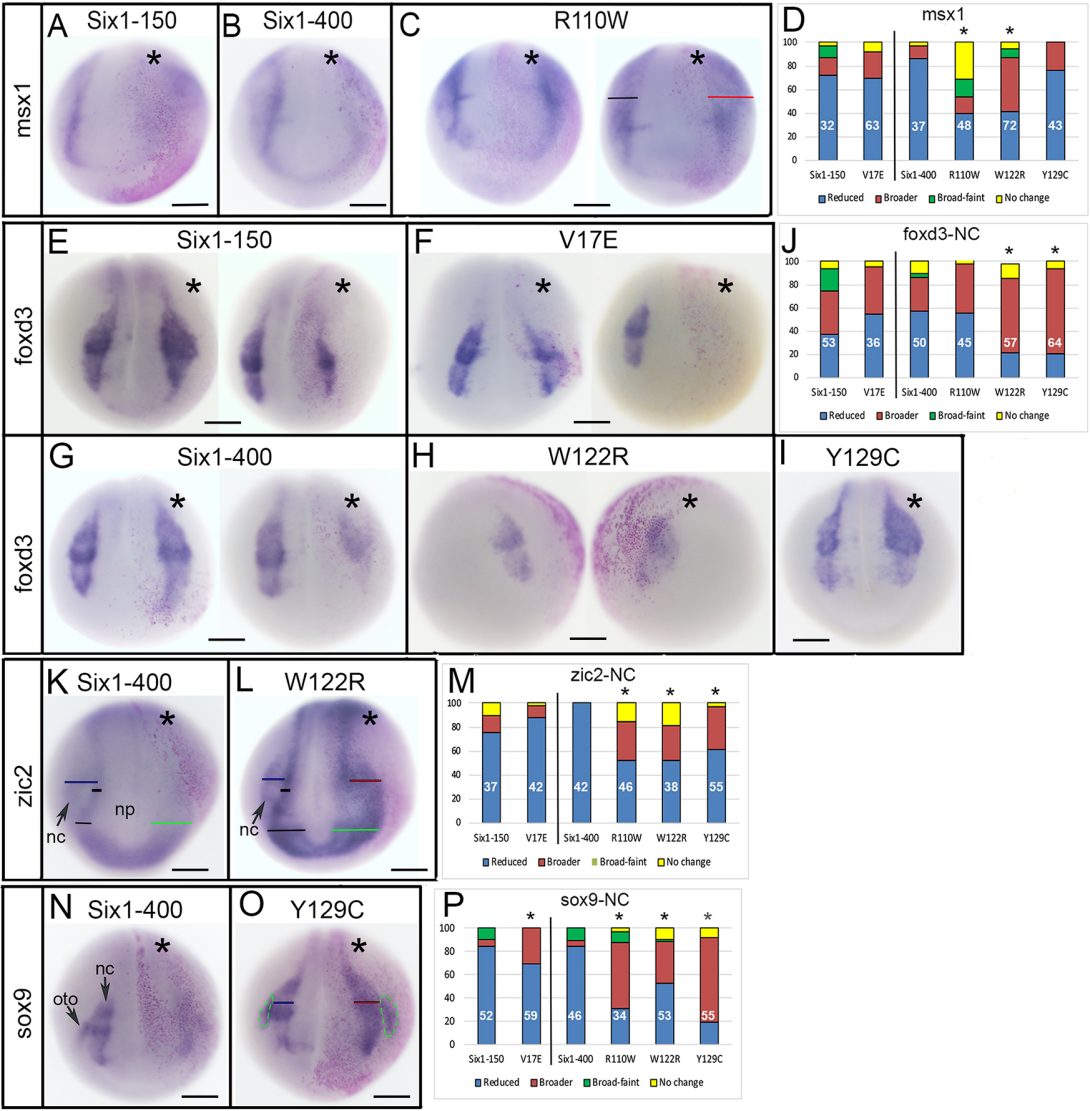
**Changes in neural border** **and neural crest gene expression.** (A,B) Both Six1WT-150 and Six1WT-400 reduce the neural border expression of *msx1* on the injected side (indicated by asterisks, pink lineage tracer). (C) R110W either did not change the *msx1* domain (left embryo) or caused it to be broader (right embryo, red bar) compared to the control side (black bar). (D) The expression domain size of *msx1* on the *Six1*-mutant-injected side was compared to the control side of the same embryo and scored as reduced (blue), broader (red), broader but fainter (green) or unchanged (yellow). Phenotypes are expressed as frequencies and the sample size is within each bar (white numbers); experiments were repeated a minimum of three times. Frequencies for Six1 mutants were compared to those for embryos injected with *Six1* WT mRNA; V17E was compared to Six1WT-150, and the others were compared to Six1WT-400. Significant differences between mutant and WT frequencies were assessed by the Chi-squared test (*P<0.05). (E) Six1WT-150 could either broaden (left embryo) or reduce (right embryo) the *foxd3* domain. (F) V17E could either broaden (left) or reduce (right) the *foxd3* domain. (G) Six1WT-400 could either broaden (left) or reduce (right) the *foxd3* domain. (H,I) W122R (H) and Y129C (I) predominantly broadened the *foxd3* domain. (J) Quantitation of *foxd3* neural crest (NC) phenotypes, as in D. (K) Six1WT-400 broadened the anterior neural plate (np) domain (green bar) of *zic2*, but reduced its neural crest (nc) domain (compare to black bars and blue bar). (L) W122R caused both the anterior neural plate domain (green bar) and neural crest domain (red bar) of *zic2* to broaden (compare to black and blue bars on control side). (M) Quantitation of *zic2* neural crest phenotypes, as in D. (N) Six1WT-400 reduced both the neural crest and otic placode (oto) domains of *sox9*. (O) Y129C broadened both the neural crest (red bar) and otic placode (green dashed lines) domains of *sox9*. (P) Quantitation of *sox9* neural crest phenotypes, as in D. Scale bars: 300 μm.

**Fig. 3.**
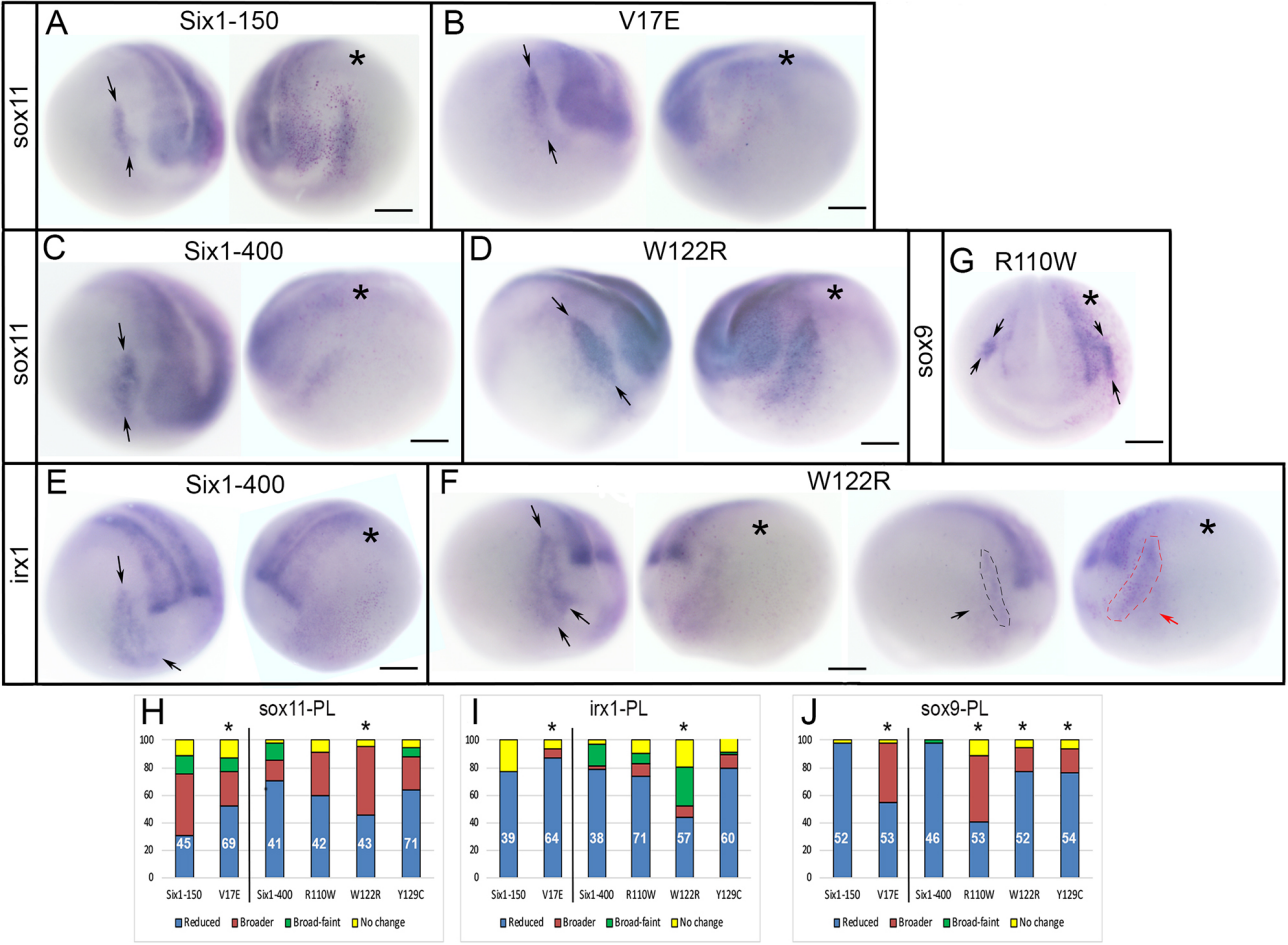
**Changes in PPE and cranial placode gene expression.** (A) Six1WT-150 expanded the *sox11* PPE domain [between arrows on control (left) side of the same embryo]. (B) V17E predominantly reduced the *sox11* PPE domain [between arrows on control (left) side of the same embryo]. (C) Six1WT-400 (right) reduced the *sox11* PPE domain [between arrows on control (left) side of the same embryo]. (D) W122R predominantly broadened the *sox11* PPE domain [between arrows on control (left) side of the same embryo]. (E) Six1WT-400 reduced the *irx1* placode domain [between arrows on control (left) side of the same embryo]. (F) W122R either caused *irx1* PPE domain (between arrows in leftmost, control image) to be broader but fainter (left embryo) or simply broader (red dashed line and red arrow in right embryo, compared to black dashed line and black arrow on control side). (G) R110W expanded *sox9* expression in the otic placode (between arrows) compared to control (left) side. Asterisks in A-G indicate the injected side. Scale bars: 300 µm. (H-J) Quantitation of *sox11* (H), *irx1* (I) and *sox9* (J) cranial placode (PL) phenotypes, as in Fig. 2D. *P<0.05.

**Fig. 4.**
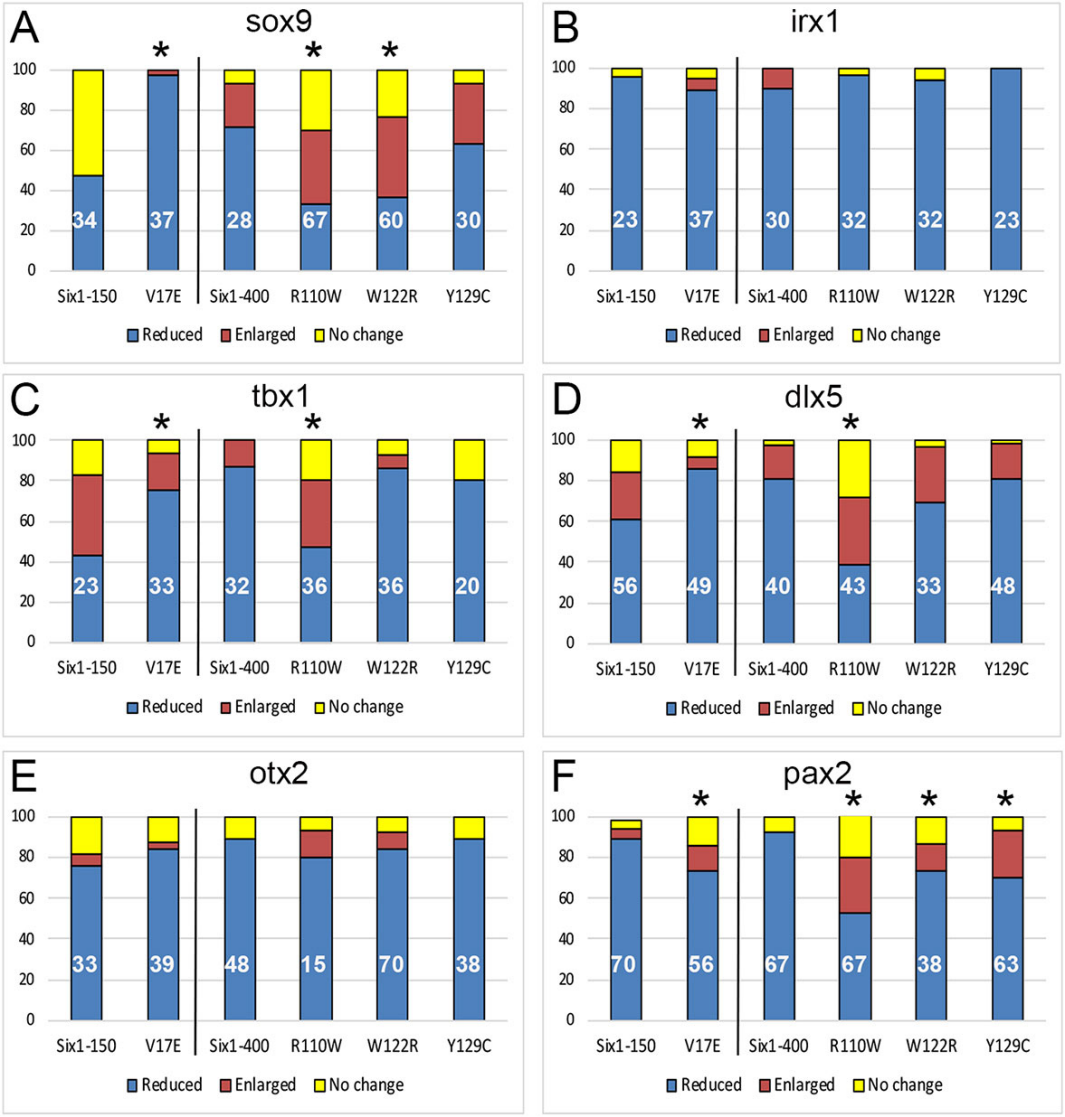
**Frequencies of otic vesicle gene expression changes.** (A) *sox9*. (B) *irx1*. (C) *tbx1*. (D) *dlx5*. (E) *otx2*. (F) *pax2*. Quantitation as described in Fig. 2D. *P<0.05.

BOS/BOR is an autosomal dominant syndrome in which patients express one WT allele and one mutated allele. Therefore, to provide a combination of WT:mutant protein we expressed *Six1* mutant mRNAs in a WT background. However, mRNAs were injected on only one side of the embryo so that the uninjected side could serve as an internal control in each animal. We also analyzed embryos injected with two different doses of *Six1* WT mRNAs (150 pg, 400 pg) to assess whether the same amount of mutant protein had similar or different effects compared to Six1WT. Because we found that *V17E* mRNA caused early lethality at >150 pg/blastomere, its effects were compared to 150 pg/blastomere of *Six1WT* mRNA (*Six1WT-150*). The other *Six1* mutants, which did not cause lethality, were expressed at 400 pg/blastomere and compared to 400 pg/blastomere of *Six1WT* mRNA (*Six1WT-400*), a dose used in numerous previous publications to determine the early developmental function of Six1 (Brugmann et al., 2004; Schlosser et al., 2008; Sullivan et al., 2019)*.* Expression of either mutant or WT Six1 caused variable changes in gene expression (reduced, broader or no change) in nearly every batch of embryos; we report the frequency of each phenotype for every gene assayed in the graphs in Figs 2-4. For simplicity, in the text we note which phenotype is the most frequent. Statistical comparison of the frequencies of phenotypes caused by expression of Six1WT versus Six1 mutants allows one to determine whether the mutation has changed the activity of the protein.

Neural border genes are required for the subsequent formation of the neural crest and pre-placodal ectoderm (PPE) (Groves and LaBonne, 2014; Saint-Jeannet and Moody, 2014; Grocott et al., 2012). Therefore, we first evaluated the effect of *Six1* mutants on *msx1*, a neural border-specifying gene that controls the expression of neural crest-specifying genes (Meulemans and Bronner-Fraser, 2004; Monsoro-Burq et al., 2005). Both *Six1WT-150* and *V17E* caused the *msx1* domain to be smaller or reduced in intensity on the injected side of the majority of embryos (Fig. 2A,D); the frequencies were not significantly different (*P*>0.05), indicating that V17E had the same activity as Six1WT on this target. *Six1WT-400* also caused a decrease in the *msx1* domain (Fig. 2B,D). However, both *R110W* and *W122R* showed this decrease significantly less frequently than WT, and also caused the domain to be broader more frequently (Fig. 2C,D); here, and in descriptions below, broader domains were either similar in intensity or reduced in intensity compared to the control side of the same embryo. In contrast, *Y129C* showed a decrease in *msx1* expression at the same frequency as *Six1WT-400* (Fig. 2D). These results indicate that, in the embryo, V17E and Y129C have about the same effect on a neural border gene as Six1WT, whereas R110W and W122R decrease the *msx1* domain less frequently and broaden it more frequently compared to Six1WT.

Since neural crest and PPE expression domains are influenced by the previous expression of neural border genes (Meulemans and Bronner-Fraser, 2004; Monsoro-Burq et al., 2005), we expected the Six1 alteration of the *msx1* expression domain to affect both. Both Six1WT-150 and V17E could either broaden or reduce the domain of *foxd3*, a neural crest specifier (Meulemans and Bronner-Fraser, 2004), and the frequencies of these effects were similar (Fig. 2E,F,J). Six1WT-400 also caused both effects (Fig. 2G,J). R110W effects were not different from those of WT (Fig. 2J), whereas W122R and Y129C caused a significantly higher frequency of *foxd3* domain expansion (Fig. 2H,I,J). *zic2* is also expressed in the neural crest domain and is required for neural crest development (Elms et al., 2003; Warner et al., 2003; Teslaa et al., 2013). In most cases, Six1WT-150 and V17E reduced *zic2* and at similar frequencies (Fig. 2M). Six1WT-400 reduced neural crest *zic2* in every embryo (Fig. 2K,M), whereas R110W, W122R and Y129C each did so at a significantly lower frequency, and in many cases made the domain broader (Fig. 2L,M). In *Xenopus*, *sox9* is expressed in both the neural crest and otic placodes (Park and Saint-Jeannet, 2010; Lee and Saint-Jeannet, 2011). In most cases, Six1WT-150 caused reduced neural crest expression of *sox9*, as did V17E, albeit at a significantly lower frequency (Fig. 2P). In most cases, Six1WT-400 also caused reduced neural crest expression of *sox9* (Fig. 2N,P); in contrast, R110W, W122R and Y129C caused reduced *sox9* at significantly lower frequencies and expanded it at significantly higher frequencies (Fig. 2O,P). Thus, compared to WT, each Six1 mutant has different effects on three genes expressed in the early neural crest: V17E was less effective than WT at reducing *sox9*; W122R and Y129C were less effective than WT at reducing *foxd3* and tended to broaden it; and R110W, W122R and Y129C were less effective than WT at reducing *zic2* and *sox9* and tended to broaden them.

As previously reported, in most cases, Six1WT-150 expanded the PPE expression of *sox11* (Sullivan et al., 2019) (Fig. 3A,H). In contrast, V17E reduced it significantly more frequently (Fig. 3B,H). In most cases, Six1WT-400 reduced *sox11*, as did R110W and Y129C, but W122R more frequently caused expansion (Fig. 3C,D,H). As previously reported (Sullivan et al., 2019), Six1WT-150 caused reduction of *irx1* in the PPE, as did V17E, albeit at a significantly higher frequency (Fig. 3I). Six1WT-400 also reduced *irx1* in the PPE, as did R110W and Y129C (Fig. 3E,I). In contrast, W122R caused a reduced *irx1* PPE domain at a significantly lower frequency and often caused expansion (Fig. 3F,I). Six1WT-150 reduced otic placode expression of *sox9*, whereas V17E reduced *sox9* at a significantly lower frequency and often broadened it (Fig. 3J). In most cases, Six1WT-400 also reduced *sox9* otic placode expression (Figs 2N and 3J), whereas R110W, W122R and Y129C each reduced *sox9* otic placode expression significantly less frequently than WT and in some cases broadened its domain (Figs 2O and 3G,J). Thus, compared to WT, each Six1 mutant protein has different effects on three placode genes: they were less effective than Six1WT (V17E broadened *sox11* less frequently; W122R reduced *irx1* less frequently), more effective than Six1WT (V17E reduced *irx1* more frequently) or caused the opposite phenotype in a subset of embryos (V17E, R110W, W122R and Y129C for *sox9*).

### Six1 mutants alter otic vesicle gene expression

**Fig. 5.**
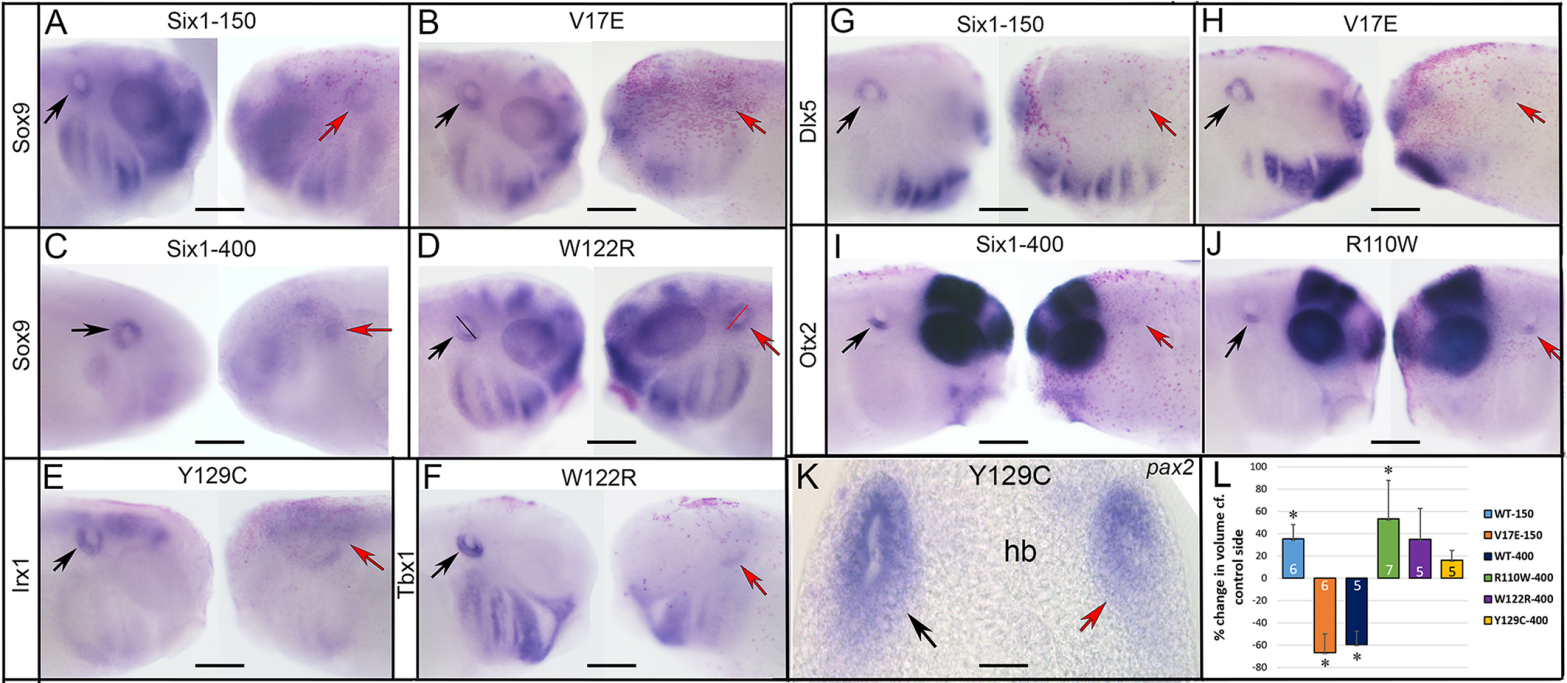
**Examples of changes in otic vesicle gene expression.** (A) Six1WT-150 reduced the otic expression of *sox9* (red arrow) compared to the control side (black arrow) of same embryo. (B) V17E had a similar effect. (C) Six1WT-400 reduced *sox9* otic expression. (D) W122R caused slightly darker otic expression of *sox9* and what appeared to be a slightly larger otic vesicle (red bar) compared to the control side (black bar). (E) Y129C reduced *irx1* otic expression. (F) W122R reduced *tbx1* otic expression. (G) Six1WT-150 reduced *dlx5* otic expression. (H) V17E also reduced *dlx5* otic expression. (I) Six1WT-400 reduced the ventral otic expression of *otx2*. (J) R110W did the same. See Fig. 4 for frequencies. (K) Some larvae were sectioned to measure otic vesicle volume. In the shown Y129C larva, *pax2* expression was reduced in the otic vesicle on the injected side (red arrow) compared to the control side (black arrow). hb, hind brain. (L) The otic vesicle volumes of SixWT, mutant Six1 and the control side of the same larva were calculated (Table S1). Because larvae were different sizes, mean experimental volumes were plotted as percentage change compared to mean control volumes (±s.e.m.) (two-tailed Student's *t*-test, *P<0.05). Six1WT-150 and R110W caused a significant increase in otic vesicle volume compared to the control side of the same embryo, whereas V17E and Six1WT-400 caused a significant decrease. Experiments were replicated three times and the number of tadpoles analyzed noted within each bar. Scale bars: 300 μm (A-J), 50 μm (K).

To assess whether these changes in early gene expression impact the initial formation of the inner ear, we raised similarly injected embryos to otic vesicle stages and assessed the expression of several transcription factors required for inner ear development (Groves and Fekete, 2012; Saint-Germain et al., 2004). Six1WT-150 reduced *sox9* otic expression in about half of the embryos (Figs 4A and 5A), whereas V17E caused a significantly greater frequency of the reduced phenotype (Figs 4A and 5B). Six1WT-400 also reduced *sox9* otic expression (Figs 4A and 5C); Y129C caused the same at a similar frequency (Fig. 4A), whereas R110W and W122R reduced expression significantly less frequently and more frequently caused expansion of the otic vesicle (Figs 4A and 5D). *irx1* was reduced by Six1WT and at similar frequencies by each mutant (Figs 4B and 5E). *tbx1* was both reduced and enlarged by Six1WT-150, whereas in most cases V17E caused reduction (Fig. 4C). In most cases, Six1WT-400 reduced *tbx1*, as did W122R and Y129C at a similar frequency (Figs 4C and 5F); in contrast, R110W reduced *tbx1* at a significantly lower frequency and expanded it more frequently (Fig. 4C). In most cases, *dlx5* was reduced by Six1WT-150 (Figs 4D and 5G); the frequency of *dlx5* reduction was significantly greater with V17E (Figs 4D and 5H). In most cases, *dlx5* was reduced by Six1WT-400, W122R and Y129C at a similar frequency; *dlx5* reduction was significantly less frequent with R110W and more embryos showed expansion (Fig. 4D). *otx2* was reduced by both levels of Six1WT, and each mutant reduced it at a similar frequency to Six1WT (Figs 4E and 5I,J). Whereas *pax2* expression was dramatically reduced by both levels of Six1WT, all four mutants caused reduction at a significantly lower frequency as well as a low frequency of expansion (Figs 4F and 5K).

Because the reduction in gene expression assayed in whole-mount *in situ* hybridization (ISH) could result from either reduced gene expression or loss of otic vesicle tissue, we sectioned a subset of the larval heads and calculated the volume of control and injected otic vesicles in the same embryo (Fig. 5K; Table S1; two-tailed paired Student's *t*-test). Six1WT-150 otic vesicle volumes were significantly larger than the otic vesicle volumes on the control side of the same embryo, whereas V17E otic vesicle volumes were significantly smaller than those on the control side (Fig. 5L). Six1WT-400 otic vesicle volumes were significantly smaller than those on the control side of the same embryo, whereas R110W, W122R and Y129C otic vesicle volumes were slightly larger, although this only reached significance for R110W (Fig. 5L; *P*<0.05). Interestingly, using the two-tailed, unpaired Student's *t*-test, we found that the V17E otic vesicle volume (5.78×10^6^ µm^3^) was significantly smaller than that of Six1WT-150 (1.92×10^6^ µm^3^; *P*<0.05), but indistinguishable from that of Six1WT-400 (1.52×10^6^ µm^3^; *P*>0.05); this comparison suggests that a low level of V17E (150 pg) has an activity comparable to that of a higher level of Six1WT (400 pg). The otic vesicle volumes of R110W, W122R and Y129C (5.24, 5.12 and 4.48×10^6^ µm^3^, respectively) each were significantly larger than that of Six1WT-400 (1.52×10^6^ µm^3^; *P*<0.05), but indistinguishable from that of Six1WT-150 (5.78×10^6^ µm^3^; *P*>0.05); this comparison suggests that a 400 pg dose of these mutant proteins has a weaker activity than the comparable level of Six1WT.

Together, these analyses demonstrate that all four Six1 mutants affect both otic vesicle volume and the expression of a subset of genes that are required for otic specification and patterning in ways that are not identical to Six1WT. Their effects are likely due to the mutations rather than overexpression because each is different from the comparable level of Six1WT.

### Six1 mutants alter ear morphology

**Fig. 6.**
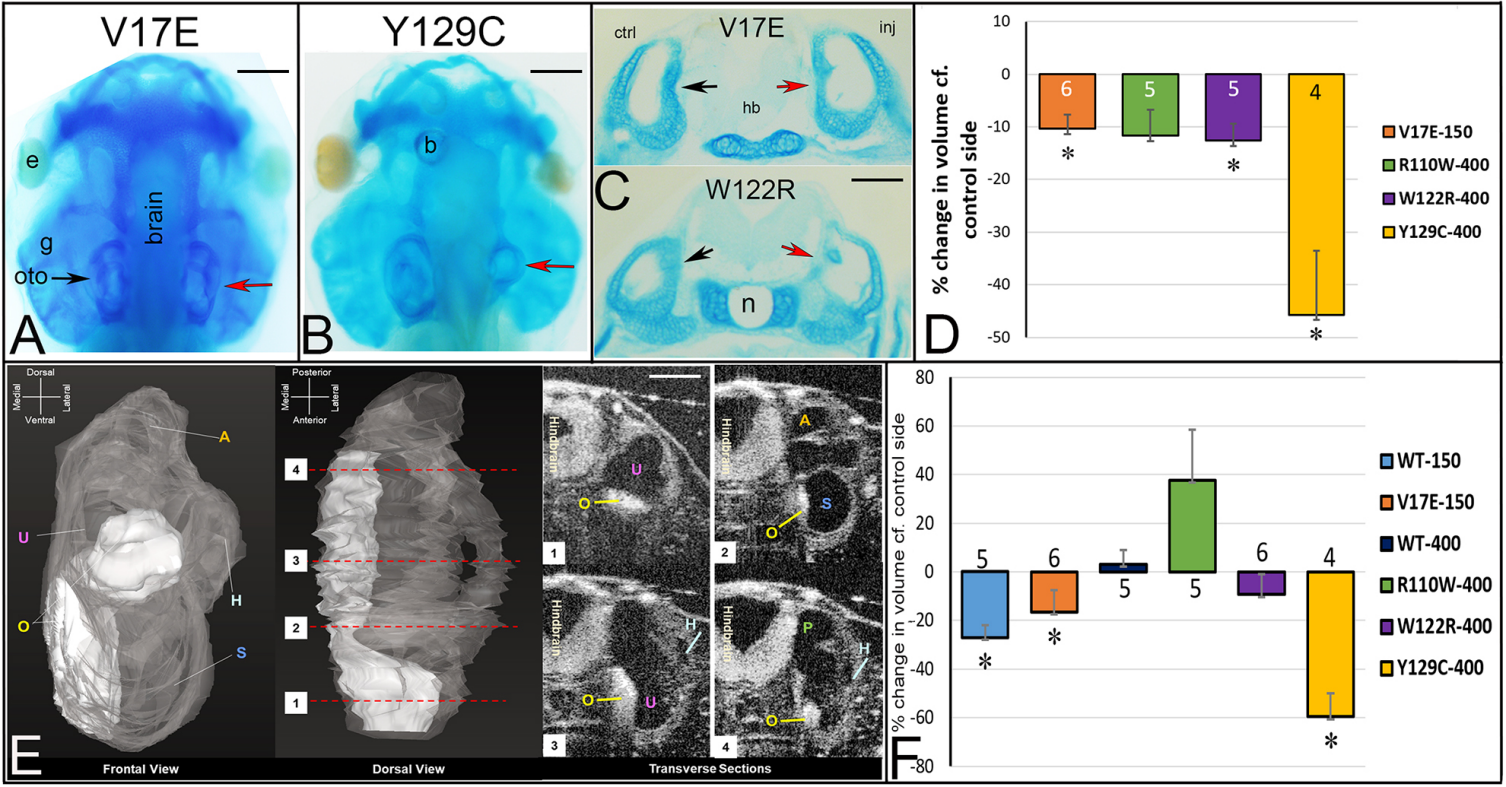
**Mutant Six1 proteins affect otic capsule and otolith volumes.** (A) Alcian Blue-stained tadpole head in which the right side expressed V17E. The otic capsule (oto) on the injected side (red arrow) is not notably different from that on the control side (black arrow) in this individual. e, eye; g, gill cartilages. Similar results were seen for R110W and W122R. (B) Alcian Blue-stained tadpole head in which the right side expressed Y129C. The otic capsule on the injected side (red arrow) is much smaller than that on the control side (left). b, bubble in the mounting medium. (C) Vibratome section reveal the cartilaginous otic capsules on control (ctrl, black arrow) and injected (inj, red arrow) sides of a V17E tadpole (top) and W122R tadpole (bottom). hb, hind brain; n, notochord. (D) The otic cartilage volumes of mutant Six1 and control sides of the same tadpole were calculated (Table S2) and compared by a paired, two-tailed Student's *t*-test. Because tadpoles were different sizes, the mean experimental volumes were plotted as percentage change compared to mean control volumes (±s.e.m.). V17E, W122R and Y129C caused significant decreases in otic cartilage volume (*P<0.05). (E) Three-dimensional reconstruction of transverse sections collected using OCT. Otic vesicle is in gray with otoliths in white from frontal (leftmost) and dorsal (middle left) views. Four transverse sections (1-4) taken at the levels indicated on the dorsal view reveal internal structures: A, anterior canal; H, horizontal canal; O, otolith; P, posterior canal; S, saccule; U, utricle. (F) Otolith volumes of SixWT, mutant Six1 and control sides of the same tadpole were calculated from OCT images (Table S3) and compared by a paired, one-tailed Student's *t*-test. As in D, the mean experimental volumes were plotted as percentage change compared to mean control volumes (±s.e.m.). Six1WT-150, V17E and Y129C resulted in significantly smaller otolith volumes (*P<0.05). Experiments were replicated three times and the number of tadpoles analyzed noted within each bar in D and F. Scale bars: 100 μm (A,B), 70 μm (C,E).

To determine whether the changes reported above affect the morphology of the inner ear and surrounding, neural crest-derived otic cartilage, we performed injections as above and fixed tadpoles at stages when the auditory and vestibular regions, including hair cell sensory patches are recognizable (Bever et al., 2003; Quick and Serrano, 2005). We then used several techniques to assess aspects of ear morphology. To reveal the cartilaginous otic capsule that is derived from cranial neural crest, heads were stained with Alcian Blue. For V17E, R110W and W122R, there were subtle asymmetries between the otic capsule on the control versus injected sides (Fig. 6A and data not shown); the Y129C otic capsule showed more obvious asymmetries than the other mutants (Fig. 6B). To quantitate these changes, heads were sectioned, and the volume of the otic cartilage on each side was calculated (Table S2) and tested for whether the experimental volume was different from that on the control side (two-tailed paired Student's *t*-test). V17E, W122R and Y129C resulted in a significantly smaller otic cartilage volume compared to the control sides (Fig. 6C,D).

In bright-field examination of live tadpoles, in which the heads are transparent, we noticed that in some specimens the otoliths of the saccule and utricle appeared reduced. Because otolith volumes are difficult to assess in fixed specimens, we used optical coherence tomography (OCT) imaging, which uses differential light interference to detect morphology in living specimens, to test whether, like otic cartilage, otolith volumes were reduced by mutant Six1 proteins (one-tailed paired Student's *t*-test). The fluid-filled lumen of the living inner ear appears dark whereas the mineralized otoliths, which reflect light, appear bright (Fig. 6E). Upon calculating otolith volumes (Table S3), we found that the mean otolith volumes were significantly smaller in Six1WT-150, V17E and Y129C inner ears compared to those on the control sides of the same tadpoles (Fig. 6F). We compared the mean volumes of the mutant otoliths to those of Six1WT otoliths to determine whether the mutant protein reduced this structure (one-tailed unpaired Student's *t*-test). Otoliths of V17E (7.2×10^5^ µm^3^) were similar in volume to those of Six1WT-150 (1.6×10^6^ µm^3^; *P*>0.05). R110W (1.8×10^6^ µm^3^) otoliths were significantly larger compared to Six1WT-400 otoliths (6.6×10^5^ µm^3^; *P*<0.05), and Y129C otoliths (1.0×10^5^ µm^3^; *P*<0.05) were significantly smaller than Six1WT-400 otoliths; W122R otoliths (1.2×10^6^ µm^3^; *P*>0.05) were not different. Thus, two of the four mutants have effects that are significantly different from those of Six1WT.

**Fig. 7.**
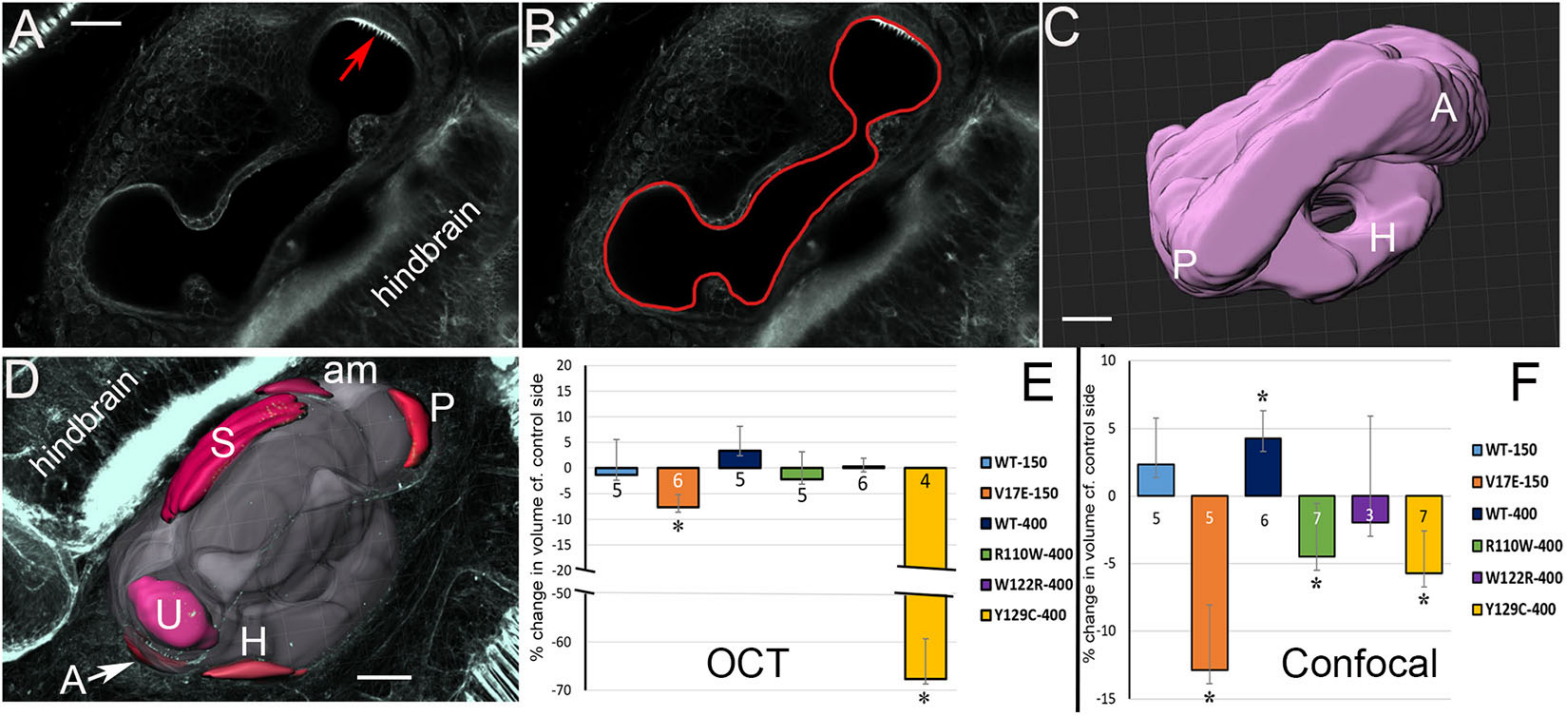
**Mutant Six1 proteins affect inner ear luminal volumes.** (A) Single confocal optical section through a phalloidin-stained tadpole inner ear showing the lumen and a single sensory patch containing hair cells (red arrow). (B) Image of same section showing outline of lumen in IMARIS software. (C) Dorsal view of a 3D reconstruction of the same inner ear, showing anterior (A), posterior (P) and horizontal (H) semicircular canals. (D) Ventral view of a 3D reconstruction of an inner ear, highlighting the different sensory end organs: A, anterior canal; H, horizontal canal; P, posterior canal; S, saccule; U, utricle. In this specimen, the amphibian papilla (am) also has differentiated, but since this was not a consistent feature at this developmental stage, it was not included in the volume measurements. (E) The inner ear luminal volumes of SixWT, mutant Six1 and control sides of the same tadpole were calculated from OCT images (Table S4) and compared by a paired, one-tailed Student's *t*-test. As in Fig. 6D, the mean experimental volumes were plotted as percentage change compared to mean control volumes (±s.e.m.). Significantly smaller volumes were detected for V17E and Y129C (*P<0.05). (F) The inner ear luminal volumes of SixWT, mutant Six1 and control sides of the same tadpole were calculated from confocal images (Table S5) and compared by a paired, one-tailed Student's *t*-test. As in Fig. 6D, the mean experimental volumes were plotted as percentage change compared to mean control volumes (±s.e.m.). Six1WT-400 caused a significant increase in luminal volume, whereas V17E, R110W and Y129C caused significantly smaller volumes (*P<0.05). Experiments were replicated three times and the number of tadpoles analyzed noted within each bar in E and F. Scale bars: 25 μm.

In some BOS/BOR patient reports, the vestibular and/or cochlear ducts are reported to be hypoplastic. To determine whether the Six1 mutants also caused a reduced luminal volume, the lumen of the inner ear labyrinth was traced from OCT images of living tadpoles (Fig. 6E). Six1WT or Six1-mutant-expressing labyrinths contained all tadpole stage vestibular and auditory structures in the appropriate position/orientation, although loss of some interior cartilage structure was noted in some Y129C inner ears stained with Alcian Blue (Fig. 6B). Luminal volumes obtained from live tadpoles by OCT were calculated (Table S4) and compared (one-tailed paired Student's *t*-test); those for V17E and Y129C were significantly smaller compared to control sides (Fig. 7E). Using a one-tailed unpaired Student's *t*-test, we found that the mean luminal volumes of V17E (7.4×10^6^ µm^3^) were significantly smaller than those of Six1WT-150 (2.1×10^7^ µm^3^; *P*<0.05). Compared to the lumens of Six1WT-400 (6.0×10^6^ µm^3^), R110W lumens were significantly larger (1.8×10^7^ µm^3^; *P*<0.05), Y129C lumens were significantly smaller (6.6×10^5^ µm^3^; *P*<0.001) and W122R lumens were not different (1.4×10^7^ µm^3^; *P*>0.05). Thus, three of the four mutants have effects that are significantly different from those of Six1WT.

To obtain a higher-resolution measurement of luminal volume, fixed tadpole heads were stained with phalloidin to reveal the luminal border. Confocal *z*-stacks were generated through the entire depth of the inner ear on both the injected and control side (Fig. 7A), the luminal surface traced in each stack (Fig. 7B), and the luminal volume calculated, compared (Table S5; one-tailed paired Student's *t*-test) and displayed in 3D (Fig. 7C). Concordant with OCT imaging of live tadpoles, fixed specimens of Six1WT and Six1 mutant labyrinths contained all structural elements (Fig. 7C). Luminal volumes of Six1WT-400 were significantly increased compared to the control sides (Fig. 7F), whereas three of the Six1 mutant proteins (V17E, R110W, Y129C) caused a significant decrease in luminal volume (Fig. 7F). In these fixed specimens, we did not observe significant differences between Six1WT effects and Six1 mutant effects on mean luminal volume (one-tailed unpaired Student's *t*-test; *P*>0.05).

Each of the compartments of the inner ear contain a sensory end organ containing hair cells, which have stereocilia that stain intensely with phalloidin, making them readily recognizable in the *z*-stacked images (Fig. 7A). Because loss of Six1 affects the differentiation of the hair cells (Bricaud and Collazo, 2006; Ahmed et al., 2012; Zhang et al., 2017), we also measured the volumes of five of the sensory patches that are well developed in the stage 47 tadpole (anterior, posterior, horizontal, saccule, utricle; Fig. 7D). For Six1WT-150 inner ears, only the posterior canal sensory patches were significantly different from those on the control side (larger) (*P*<0.05; Fig. S1), and for Six1WT-400 inner ears, only the utricle sensory patches were significantly different from those on the control side (larger) (*P*<0.05; Fig. S1). For the Six1 mutant inner ears, only Y129C caused significantly different volumes: the posterior canal and horizontal canal sensory patches were significantly smaller than those on the control side (*P*<0.05; Fig. S1). V17E sensory patch volumes were not significantly different from those of Six1WT-150 (Fig. S2A). Compared to Six1WT-400 (Fig. S2B-F), only Y129C caused significantly smaller posterior canal (WT 1.8×10^5^ vs 7.4×10^4^ µm^3^; *P*<0.05) and utricular (WT 1.6×10^5^ vs 1.0×10^5^ µm^3^; *P*<0.05) sensory patches. These latter comparisons indicate that two of the four mutants have effects on some sensory patches that are different from Six1WT effects.

Together, these morphological measurements show that otic capsule and several aspects of inner ear morphology are disrupted by each BOS/BOR Six1 mutation in ways that differ from overexpression of the WT protein, and that those caused by Y129C were the most consistently significant.

## DISCUSSION

It is important to decipher the consequences of mutated SIX1 on the development of the involved tissues to understand the function of the altered proteins as well as the underlying causes of the BOS/BOR phenotypes. These include the external ear (pinna deformities, preauricular pits), middle ear (ossicle defects), inner ears (vestibular and cochlear aqueducts), second branchial arch (fistulas/cysts) and kidney (Smith, 2018). Many of these phenotypes contribute to the characteristic hearing loss. Several studies have attempted to categorize patient phenotypes according to the mutation in *EYA1* or *SIX1*, but phenotypes are so variable, even within the same family, that no significant correlations were found (Ceruti et al., 2002; Kemperman et al., 2002; Ruf et al., 2003, 2004; Ito et al., 2006; Sanggaard et al., 2007; Kochhar et al., 2008; Krug et al., 2011). One contributing factor to the inability to sort phenotypes according to genotypes is that the clinical reports often are not sufficiently detailed; they rarely use magnetic resonance imaging to describe inner and middle ear defects or ultrasound to detect renal defects. Another potential contributor to variability is that early gene expression changes may subtly alter the regulatory networks controlling the differentiation of the affected tissues. To address this, we assessed whether four different mutations found in *SIX1* affect different aspects of craniofacial embryogenesis: early ectodermal gene expression (neural border, neural crest, PPE); otic vesicle gene expression; and the morphology of the otic capsule and inner ear. We chose to study whether mutations in different regions of the protein, two in the SD (V17E, R110W) and two near or in the HD (W122R, Y129C), have different effects on one or more of these early developmental processes, and whether these effects differ from those caused by expressing Six1WT.

### The biochemical effect of SIX1 mutations

A study of BOS/BOR mutations in *EYA1* concluded that the mutated proteins act in a dominant-negative fashion rather than as hypomorphs (Li et al., 2010). Likewise, it has been proposed that mutant SIX1 proteins act in a dominant-negative manner by either competing with WT protein for DNA binding sites or competing for the EYA1 co-activator (Kochhar et al., 2007; Hoskins et al., 2007). However, since patients carry one normal *SIX1* allele and one mutated allele that is transcriptionally deficient, SIX1 mutants could simply be hypomorphic. To address this, the interactions of mutant SIX1 proteins with EYA proteins and DNA were assessed by a variety of biochemical assays in various mammalian cell lines. Patrick et al. (2009) showed that V17E does not form a complex with the protein-protein interaction domain (ED) of EYA2, does not translocate EYA2 to the nucleus and does not activate the transcription of the same MEF3 reporter we used in this study (Spitz et al., 1998). This could indicate that V17E acts as a dominant negative by binding to DNA without an Eya cofactor. However, since the presence of EYA2 was shown to stabilize all mutants tested except V17E (Patrick et al., 2009), it remains possible that V17E acts as a hypomorph. In a yeast two-hybrid assay, R110W showed a reduced interaction with the ED of Eya1 (Ruf et al., 2004), but in a different assay it bound the ED of EYA2 and transported it into the nucleus (Patrick et al., 2009). Both studies showed that although R110W can bind to DNA, it does not activate MEF3 reporter transcription either alone or in combination with Eya1/EYA2. Another consideration is that mutations in the SD could result in changing the specificity of cofactor binding, as demonstrated in the fly (Kenyon et al., 2005b). The functional deficits of W122R have not been directly studied in SIX1. However, it has been proposed that the linker region between the sixth α-helix in the SD and the HD may directly contact the minor groove upon DNA binding (Patrick et al., 2009), and therefore mutations in this region could prevent or reduce DNA binding efficiency. Y129C can interact with the ED of Eya1/EYA2 and translocate EYA2 to the nucleus, but it does not bind DNA or activate MEF3 reporter transcription (Ruf et al., 2004; Patrick et al., 2009). The Y129C mutation in zebrafish *six1a* likewise fails to drive transcription via an ARE reporter (Bricaud and Collazo, 2011). Our MEF3 reporter luciferase results for the four mutations in *Xenopus Six1* corroborate the human, mouse and fish assays, demonstrating that there is similar biochemical activity across species.

### Effects on craniofacial gene expression

Because biochemical assays indicate that the different mutant SIX1 proteins can have different binding affinities for either Eya proteins or DNA, we asked whether they would have different effects on embryonic gene expression and whether their effects differ from those of Six1WT. To accomplish this, we made four of the human mutations in *Xenopus Six1* and expressed the mutant proteins on one side of embryos to assess their effects on the expression of a large number of genes that are required for the development of precursors of the tissues affected in BOS/BOR: the neural crest, PPE and otic placode. Several studies have shown that expressing Six1WT in embryos causes changes in early ectodermal gene expression (Brugmann et al., 2004; Schlosser and Ahrens, 2004; Christophorou et al., 2009; Schlosser et al., 2008; Sullivan et al., 2019). To determine whether the mutant Six1 proteins have a different effect on gene expression from Six1WT, we compared the frequency of gene expression changes (determined by comparing to the control side of the same embryo) caused by each manipulation. We categorised the Six1 mutant protein effects as follows: (1) stronger than those of a comparable level of Six1WT, i.e. the phenotype occurred significantly more frequently; (2) weaker than those of a comparable level of Six1WT, i.e. the phenotype occurred significantly less frequently; or (3) opposite to those of Six1WT, e.g. most frequently causing a broader expression domain when Six1WT most frequently causes a smaller expression domain (Table 1).

**Table 1.**
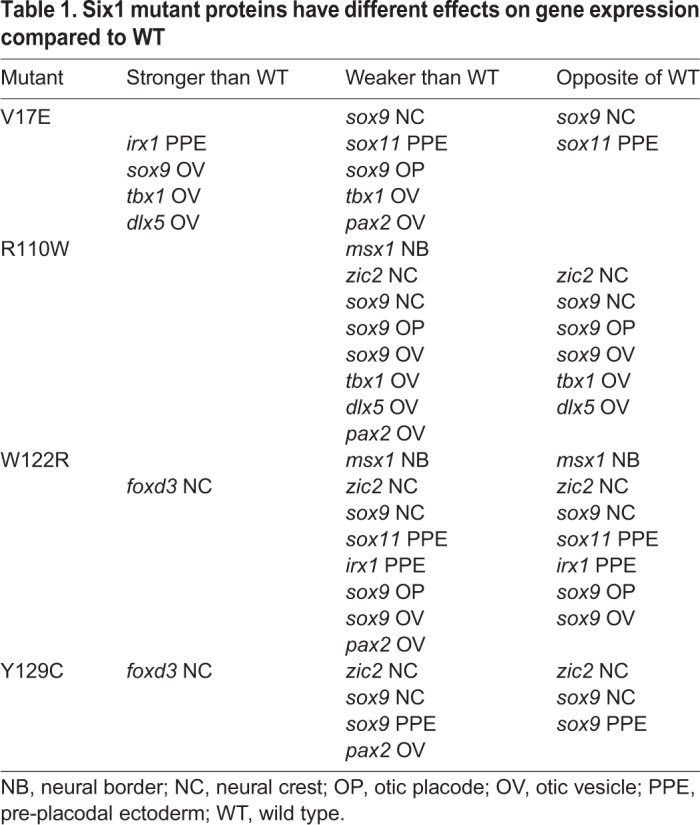
**Six1 mutant proteins** **have different effects on gene expression compared to WT**

There were a few changes that were common to all four Six1 mutants compared to comparable levels of Six1 WT: (1) a weaker effect on the neural crest and otic placode expression of *sox9*; (2) a weaker effect on *pax2* otic vesicle expression; (3) an opposite effect on *sox9* neural crest expression in a subset of embryos; and (4) no significant effects on *irx1* or *otx*2 otic vesicle expression. However, for the most part, the four Six1 mutants had effects that were distinct from each other. For example, V17E showed the largest number of stronger effects, particularly on otic vesicle genes, and the fewest number of opposite effects. Two effects (*sox9* neural crest, *sox11* PPE) were categorized as opposite because a higher frequency of embryos showed the opposite effect compared to Six1WT-150. However, the frequency of V17E effects on early gene expression were not significantly different from those of Six1WT-400 (Chi-square, *P*>0.05), except for *sox9* otic placode expression, indicating that this mutation causes the protein to act more repressive, just like high levels of the WT protein. It will be interesting to test in future experiments whether this effect is due to the lack of interaction with Eya1 and/or limited amounts of endogenous Eya1. Interestingly, overall, R110W and W122R, which are in close proximity in the protein, had many similar effects. For example, both affected *msx1*, the neural border gene, differently from Six1WT. Both affected otic vesicle genes in a dual fashion: they were repressed less than Six1WT (weaker) and expanded more (opposite). However, R110W and W122R do not show identical effects, perhaps because R110W is likely to affect partner interactions only, whereas W122R is predicted to affect both partner interactions and DNA binding. Finally, neither V17E nor R110W differentially affected *foxd3* expression, whereas both W122R and Y129C had a weaker effect on this neural crest gene.

By assessing several neural crest, PPE/cranial placode and otic vesicle genes, we revealed that each mutant showed a combination of the effects depending upon which gene was assessed, which did not segregate in strict accordance with tissue progenitor type (Table 1). For example, V17E had a stronger effect on *irx1* PPE expression but a weaker and opposite effect on *sox11* PPE expression. V17E also had a stronger effect on some otic vesicle genes and a weaker effect on others. W122R and Y129C had stronger effects on *foxd3* neural crest expression, yet weaker and opposite effects on *zic2* and *sox9* neural crest expression. One potential explanation for these different effects is tissue-specific and temporal-specific availability of cofactors that affect Six1 transcriptional activity, such as Eya1 and a large number of other potential interactors that are expressed in these tissues (Neilson et al., 2010). For example, a single-cell RNA-sequencing dataset indicates that neural crest cells do not express *Eya1*, but do express *Eya3* and *Eya4* (Briggs et al., 2018)*.* Interestingly, Mutsuddi et al. (2005) predicted that the effects of BOS/BOR mutations in EYA1 could differ from target gene to target gene by being deficient in recruiting different sets of interacting proteins to promoters and thus leading to abnormal transcriptional output. Since cofactors other than Eya1 could affect whether Six1 functions as a transcriptional repressor versus activator (Silver et al., 2003; Brugmann et al., 2004), identifying the interaction of Six1 mutant proteins with the cofactors available in each progenitor domain is an important next step. One also needs to consider whether mutant Six1 proteins alter the developmental influence of Six4, which also is expressed in placodes and binds to similar elements on the DNA (Kawakami et al., 1996, 2000). Another consideration is that the genes we monitored are likely a mixture of direct and indirect targets of Six1; Six1 could directly repress a repressor leading to activation of an indirect target. Since several large-scale screens in multiple animals have revealed many hundreds of Six1 transcriptional targets in a variety of tissues (e.g. Ando et al., 2005; Jusiak et al., 2014; Yan et al., 2015; Riddiford and Schlosser, 2016), sorting out the details of the *Six1* gene regulatory network involved in craniofacial tissue development is an important next step.

### Effects on otic morphogenesis

Several studies have described severe otic defects in *Six1*-null mice that lead to hearing loss (Laclef et al., 2003; Zheng et al., 2003; Zhang et al., 2017). While the otic vesicle forms at the appropriate developmental time, it does not undergo proper morphogenesis. In contrast, *Six1*-heterozygous inner ears, which are similar to BOS/BOR in having one normal copy of SIX1, appear to develop normally at the histological level (Laclef et al., 2003), although one mouse line was characterized by a low frequency of truncated inner ear morphology and conductive hearing loss due to middle ear defects (Zheng et al., 2003). Thus, *Six1* haploinsufficiency is mostly tolerated in inner ear development. Interestingly, an N-ethyl-N-nitrosourea (ENU)-induced mutation in mouse (E135G) that changes the second residue in the N-terminal region of the HD (residue E125 in human; Fig. 1A), showed that the presence of one copy of *Six1WT* influences the effects of the mutation (Bosman et al., 2009). Whereas the homozygous mutants are missing semicircular canals, and have fewer hair cells, VIIIg defects and malformed middle ear ossicles, the heterozygotes have only very subtle inner ear defects. This indicates that the availability of endogenous Six1WT in the presence of mutant protein abrogates the severe homozygous mutant inner ear defects. Similarly, we found that Six1 mutant protein in the presence of endogenous Six1WT disrupted gene expression in the otic placode and otic vesicle, but these changes did not severely perturb the morphology of the otic capsule or structures in the inner ear. The otic capsule, otoconia and hair cells differentiate and the structural elements were all recognizable. However, there were demonstrable effects: the volumes of the otic cartilage, otoliths, inner ear lumen and sensory end organs are variably reduced by each mutant. Interestingly, the changes in otic vesicle volume did not predict the changes in otic cartilage volume. For example, although Y129C caused enlarged otic vesicles, otic cartilage volume was significantly reduced. Similarly, although V17E caused a large reduction in otic vesicle size, the otic cartilage volume was only modestly reduced. These apparent discrepancies may be due to the otic cartilage being derived from cranial neural crest rather than the otic vesicle, and the mutations having differential effects on these two precursor populations. Another puzzling difference between early and later effects is that Y129C showed the fewest disruptions in otic placode and otic vesicle gene expression, yet it showed the greatest disruption of tadpole ear morphology, and V17E showed the most effects at otic vesicle stages but the mildest effects in the tadpole ear (Table 1). While the latter might be due to only mildly affected V17E embryos surviving to tadpole stages, alternatively, the two types of disruptions – loss of co-factor binding (V17E) versus loss of DNA binding (Y129C) – may lead to very different transcriptional roles during PPE/placode formation versus tadpole ear morphogenesis.

### Conclusions

The expression of each of the mutant Six1 proteins causes early and distinctly different gene expression disruptions that affect the progenitors of the neural crest and otic cells that ultimately give rise to the inner, middle and external ears and branchial tissues that are affected in BOS/BOR. The effects we report are highly variable within each experimental group, perhaps because we sampled a genetically diverse population derived from different outbred parents. However, similar variability in phenotypes is a hallmark of BOS/BOR (Smith, 2018). An important next step will be to discover how these varied defects that arise during embryonic stages ultimately lead to the variable patient phenotypes.

## MATERIALS AND METHODS

Many of the methods were supported by Xenbase (http://www.xenbase.org/, RRID: SCR_003280) and the National *Xenopus* Resource (http://mbl.edu/xenopus/, RRID: SCR_013731).

### Generation of Six1 mutant constructs

Four different mutations associated with BOS/BOR (Fig. 1A) were introduced into the *Xenopus laevis pCS2-Six1* plasmid (Brugmann et al., 2004) with the QuikChange Lightning Site-Directed Mutagenesis kit (Agilent). Each mutant was constructed by a single-nucleotide base change: *Six1-V17E* (GTG to GAG), *Six1-R110W* (AGG to TGG), *Six1-W122R* (TGG to AGG) and *Six1-Y129C* (TAC to TGC). All constructs were fully sequenced in both directions.

### *In vitro* synthesis of mRNAs and antisense RNA probes

mRNAs encoding *Six1WT* (Brugmann et al., 2004), each of the *Six1* mutants and a nuclear-localized *β-galactosidase* (*nβgal*) lineage tracer were synthesized *in vitro* according to the manufacturer's protocols (mMessage mMachine kit, Ambion). Antisense RNA probes for ISH were synthesized *in vitro* (MEGAscript kit; Ambion) as previously described (Yan et al., 2009).

### Obtaining embryos and microinjections

Fertilized embryos were obtained by natural mating of outbred, WT adult *Xenopus laevis* males and females as described (Moody, 2000, 2018). The breeding colony is comprised of adult, outbred *Xenopus laevis* of unknown age obtained from Nasco (Fort Atkinson, WI, USA) and maintained in compliance with The George Washington University Institutional Animal Care and Use Committee protocol A233. Embryos were chosen at the two-cell stage to accurately identify the dorsal and ventral animal blastomeres (Klein, 1987; Miyata et al., 1987; Moody, 2018). When selected embryos reached eight cells, the dorsal-animal and ventral-animal blastomeres that predominantly give rise to the neural crest and cranial placodes (Moody and Kline, 1990) were microinjected with 1 nl mRNA according to standard methods (Moody, 2000, 2018). Embryos were cultured in diluted Steinberg's solution until fixation.

### Luciferase assays

HEK293T cells (American Type Culture Collection, CRL-3216), cultured with Dulbecco's modified Eagle medium (HyClone)+10% fetal bovine serum (Gibco)+penicillin-streptomycin (Gibco), were seeded onto 24-well plates at 200,000 cells/well; 24 h later, cells were transfected using X-tremeGENE 9 DNA (Sigma-Aldrich) transfection reagent with 200 ng/well pGL3-6xMEF3-Firefly luciferase (reporter plasmid, Spitz et al., 1998), 20 ng/well TK-*Renilla* luciferase, 400 ng/well of either *pCS2*, *pCS2-Six1WT*, *pCS2-5′Myc-Eya1*, *pCS2-Six1WT* plus *pCS2-5′Myc-Eya1*, *pCS2-Six1-V17E*, *pCS2-Six1-V17E* plus *pCS2-5′Myc-Eya1*, *pCS2-Six1-R110W*, *pCS2-Six1-R110W* plus *pCS2-5′Myc-Eya1*, *pCS2-Six1-W122R*, *pCS2-Six1-W122R* plus *pCS2-5′Myc-Eya1*, *pCS2-Six1-Y129C* or *pCS2-Six1-Y129C* plus *pCS2-5′Myc-Eya1*. The pGL3-6xMEF3 luciferase reporter was previously shown to bind *Xenopus* Six1 (Ford et al., 2000). Forty-eight hours after transfection, cells were lysed with passive lysis buffer (Promega), and the resulting extracts analyzed for Firefly and *Renilla* luciferase activities using the Dual-luciferase reporter assay system (Promega). Experiments were repeated five times; after testing the data for normality (Kolmogorov–Smirnov test), a one-way ANOVA with Tukey post hoc multiple comparisons test was performed using GraphPad Prism 8 software. Expression of exogenous proteins from the transfected plasmids was confirmed by standard quantitative western blotting using primary antibodies against Six1 (D5S2S, #16960; 1:1000), Myc-tag (9B11, #2276, 1:1000) and β-actin (13E5, #4970. 1:1000) (Cell Signaling Technology, Danvers, MA, USA) and secondary antibodies (IRDye 680RD donkey anti-rabbit IgG #92568073, 1:5000; IRDye 800CW donkey anti-rabbit IgG #92532213, 1:5000; IRDye 800CW goat anti-mouse #92532219, 1:5000; LI-COR Biosciences, Lincoln, NE, USA) (A.L.P.T., unpublished).

### Nuclear staining

HEK293T cells, obtained and cultured as above, were seeded onto Nunc Lab-Tek II chamber slides (Nalge) at 400,000 cells/chamber; 24 h after plating, cells were transfected with a total of 2000 ng/chamber of *pCS2-5′Myc-Eya1* plus one of the following: *pCS2-Six1-3′FLAG*, *pCS2-Six1-V17E-3′FLAG*, *pCS2-Six1-R110W-3′FLAG*, *pCS2-Six1-W112R-3′FLAG* or *pCS2-Six1-Y129C-3′FLAG*. Forty-eight hours after transfection, cells were fixed in 4% paraformaldehyde and processed for immunostaining by standard methods using mouse anti-FLAG (9A3, #8146, 1:1600) and rabbit anti-Myc (71D10, #2278, 1:200) monoclonal antibodies (Cell Signaling Technology, Danvers, MA, USA) followed by Alexa Fluor 488-conjugated anti-rabbit (#4412, 1:1000) and Alexa Fluor 568-conjugated anti-mouse (#A1104, 1:1000) secondary antibodies, and NucBlue nuclear counterstain (#R37605) (Thermo Fisher Scientific, Waltham MA, USA). Experiments were repeated three times, and at least five fields per slide analyzed using a Zeiss LSM 710 confocal microscope. It should be noted that low levels of endogenous SIX1 mRNA and protein can be detected in the HEK293T cell line (https://www.proteinatlas.org/ENSG00000126778-SIX1/cell#rna and confirmed by A.L.P.T., unpublished).

### Histochemistry and ISH

Embryos were cultured to neural plate border (stage 13), neural plate (stages 16-18) and otocyst (stages 28-32) stages (Nieuwkoop and Faber, 1994), fixed in 4% paraformaldehyde, stained for β-Gal histochemistry, and processed for ISH as described previously (Yan et al., 2009). In embryos in which the *nβgal* lineage tracer was located in the appropriate tissue domains, the position, intensity and size of the expression domains of *dlx5*, *foxd3*, *irx1*, *msx1*, *otx2*, *pax2*, *sox9*, *sox11*, *tbx1* and *zic2* were compared between the injected, lineage-labeled side and the control, uninjected side of the same embryo, thus controlling for inter-embryo variation. Embryos for each assay were derived from a minimum of three different sets of outbred parents. Embryos were scored for gene expression changes independently by at least two of the authors, and the values reported are means of their independent scores. Gene expression changes were scored in three categories: (1) decreased expression, which could be either a smaller domain or same sized domain with reduced intensity compared to the control side of the same embryo; (2) broader expression domain, which was of the same intensity or reduced intensity compared to the control side of the same embryo; or (3) no change compared to the control side of the same embryo. The Chi-square test was used to determine if the frequency of gene expression changes were significantly different between *Six1*-mutant-injected embryos and *Six1WT*-injected embryos (*P*<0.05).

### Vibratome sections and area analysis

A subset of larvae (stages 30-34) that were processed by ISH expression were embedded in a gelatin-based medium [0.5% gelatin, 30% bovine serum albumin, 20% sucrose, hardened with glutaraldehyde (75 µl/ml)], and vibratome sectioned at 40 µm in the transverse plane. Serial section images through the otic vesicle of a minimum of six embryos per mRNA injection were collected using a Leica Tiling microscope. The area of both control and injected otic vesicles from every section per embryo was measured using Zen Blue software (Zeiss Zen 2.0) to calculate the volume. Because the larvae were of different sizes, the volume of each mutant otic vesicle was expressed as a percentage of the control vesicle of the same larva and plotted as percent change from control: [(experimental−control)/control]×100. A two-tailed paired Student's *t*-test was used to determine if the volume of the *Six1*-mutant-injected otic vesicle was significantly different from that on the control side (*P*<0.05). A two-tailed unpaired Student's *t*-test was used to determine if the volumes of *Six1*-mutant-injected otic vesicles were significantly different from those of *Six1WT*-injected otic vesicles (*P*<0.05).

### Alcian Blue staining

To assess the development of the neural-crest-derived cartilage that forms a capsule surrounding the inner ear, embryos were microinjected as above, grown to tadpole stages, fixed in 4% paraformaldehyde and stained in 0.1% Alcian Blue in acidic alcohol (70% EtOH, 0.37% HCl) according to Young et al. (2017). They were vibratome sectioned at 40 µm as above. Serial section images were collected, and the area of otic cartilage on each side of the head (injected and control) traced in every section and converted to volume using Olympus cellSens Standard software (version 1.17). Because the tadpoles were of different sizes, the otic cartilage volume of each mutant ear was plotted as percentage change, as above. A two-tailed paired Student's *t*-test was used to determine if the volume of the *Six1*-mutant-injected otic capsule was significantly different from that on the control side of the same embryo (*P*<0.05).

### OCT and volumetric analyses

Tadpoles (stages 46-47) were anesthetized in 0.01% benzocaine solution, aligned in an agar-coated dish with dorsal sides facing the OCT imaging field and live imaged with a Thorlabs Telesto series OCT Imaging System with a 1300 nm spectral domain. Images collected from living tadpoles were processed with the ThorImageOCT software (version 5.1.1). Sectional image stacks, collected every 2 µm, were exported as tiff images into Free-D 3D image reconstruction and modeling software (version 1.15; Andrey and Maurin, 2005). To measure volumes in the living tadpole, which avoids fixation artefacts, the outer boundary of each saccular and utricular otoliths and the luminal surface were traced in every other section of the injected and control inner ears of each embryo. Because the tadpoles were of different sizes, the otolith and luminal volumes of each mutant ear were expressed as percentage changes from controls, as above. A one-tailed paired Student's *t*-test was used to determine if the otolith and luminal volumes on the *Six1*-mutant-injected side were significantly smaller than those on the control side (*P*<0.05). A one-tailed unpaired Student's *t*-test was used to determine if *Six1* mutant otolith and luminal volumes were significantly smaller than those of *Six1WT* specimens (*P*<0.05).

### Whole-mount phalloidin staining and confocal image analysis

To measure the volumes of the inner ear lumens and sensory hair cell end organs, embryos were microinjected as above, grown to the same tadpole stages, fixed in 4% paraformaldehyde/PBS and stained with Alexa Fluor 568 Phalloidin (Thermo Fisher Scientific, 1:250 dilution) to detect F-actin. *Z*-stacks through the entire inner ear were acquired every 1 µm with a Zeiss LSM710 confocal microscope, and the stacked confocal images imported into IMARIS software (Bitplane version 9.2) to generate a 3D reconstruction of the inner ear. The luminal surface and the boundary of each sensory hair cell patch was traced in each optical section of the injected and control inner ears of each embryo, setting the same threshold parameters for both ears. After segmenting the image data, IMARIS calculated the surface area and volumes for each sample. Because the tadpoles were of different sizes, the luminal volume of each mutant ear was expressed as percentage change from control, as above. A one-tailed paired Student's *t*-test was used to determine if sensory patch and luminal volumes on the *Six1*-mutant-injected side were significantly different from those on the control side (*P*<0.05). A one-tailed unpaired Student's *t*-test was used to determine if they were significantly different between *Six1WT*-injected and *Six1*-mutant-injected specimens (*P*<0.05).

## Supplementary Material

Supplementary information
