# Supplementary material for: Six1 proteins with human branchio-oto-renal mutations differentially affect cranial gene expression and otic development
Source: Dis Model Mech. 2020 Mar 3;13(3):dmm043489. doi: 10.1242/dmm.043489 (PMC7063838; doi:10.1242/dmm.043489)
Supplement: Supplementary information [file dmm-13-043489-s1.pdf]

Supplemental Figure 1A

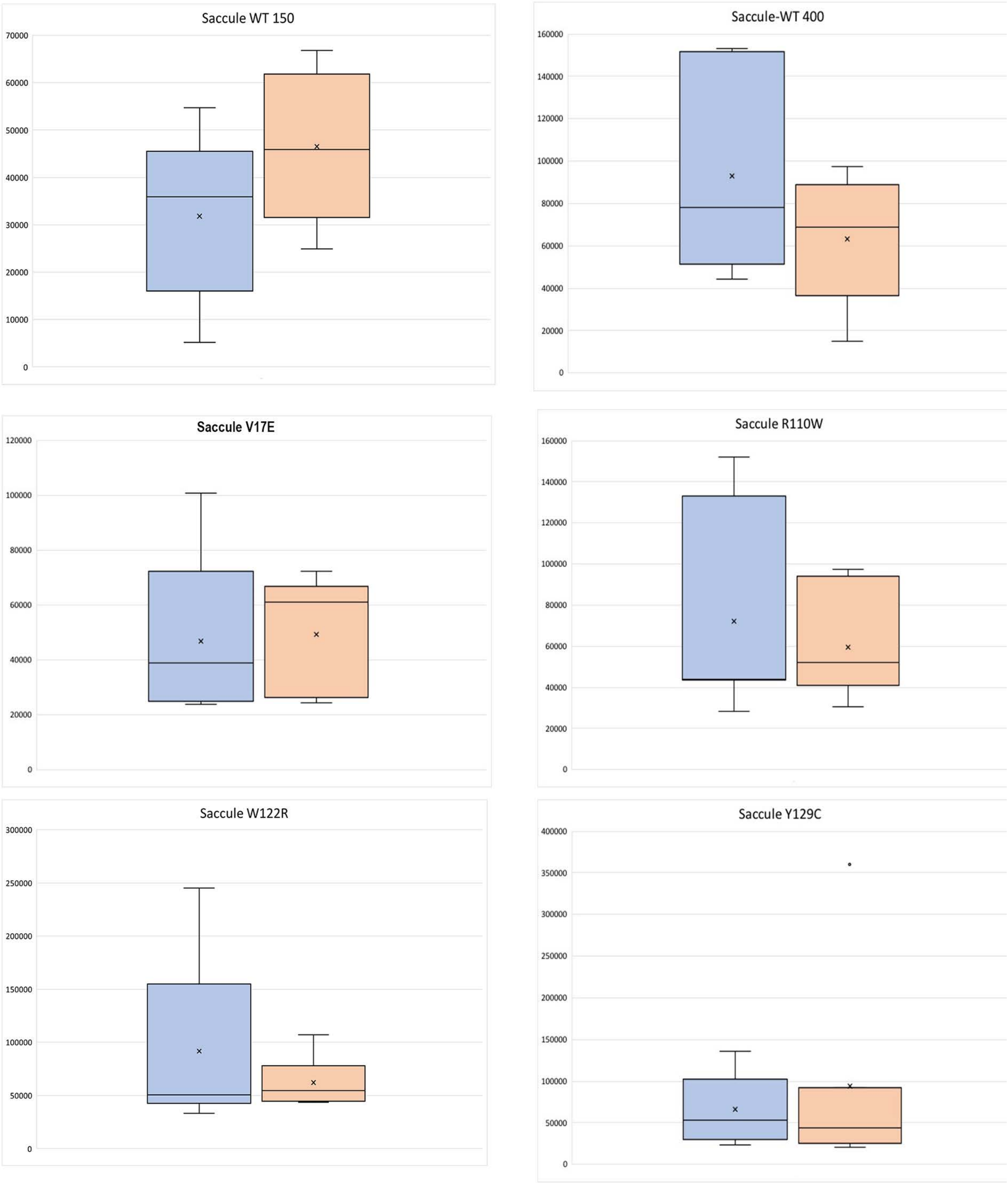

Disease Models & Mechanisms • Supplementary information

Supplemental Figure 1B

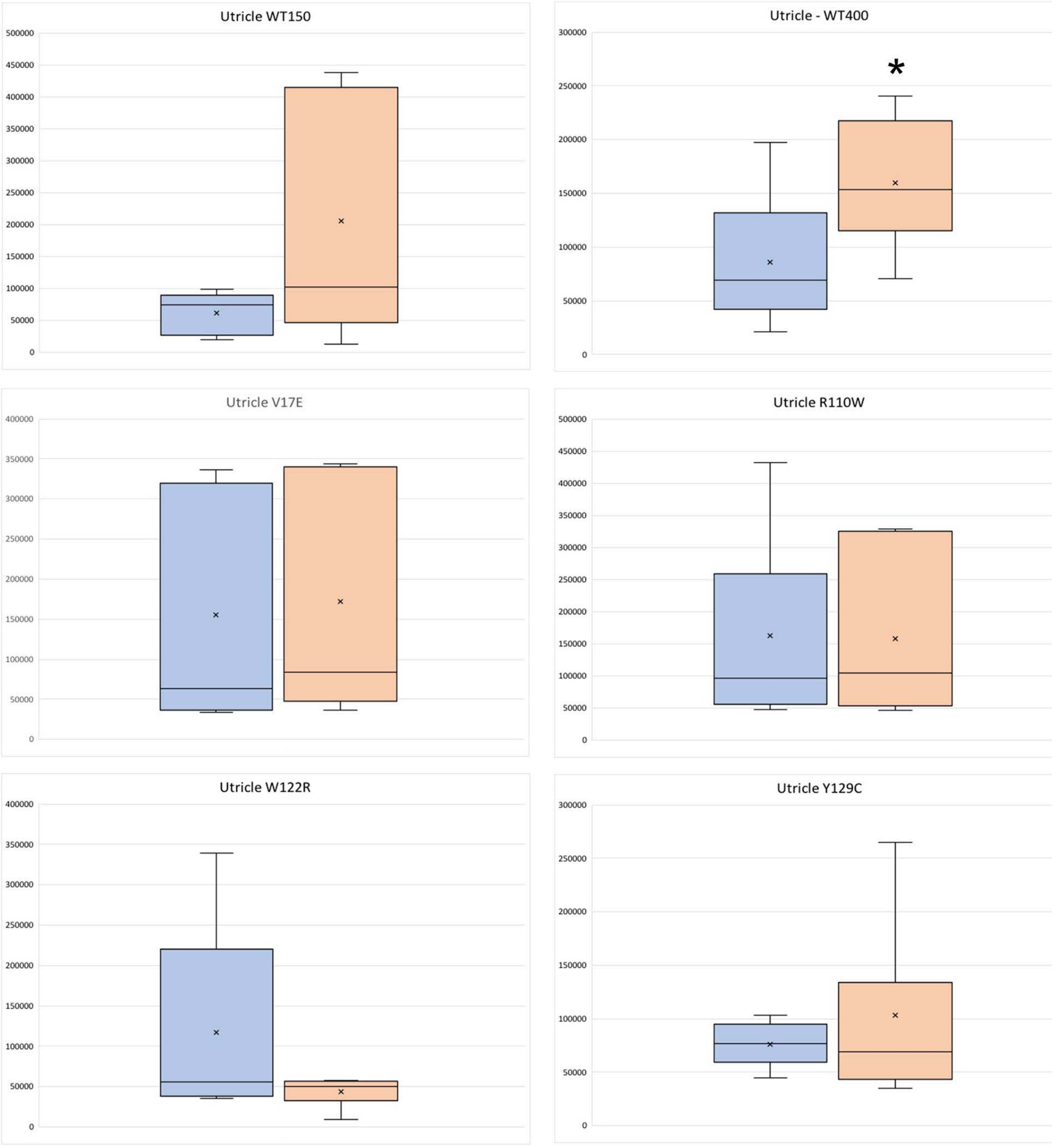

Disease Models & Mechanisms • Supplementary information

Supplemental Figure 1C

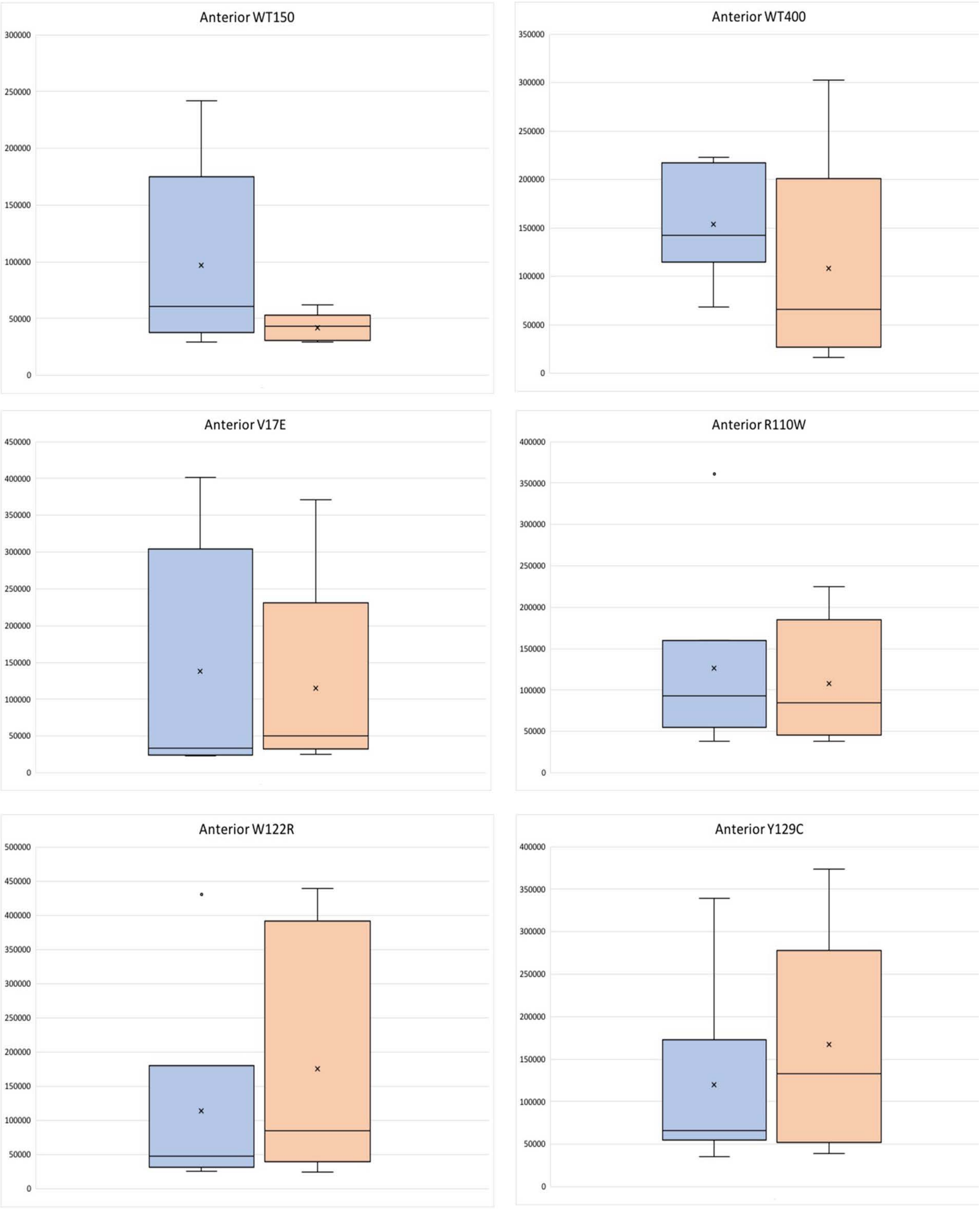

Supplemental Figure 1D

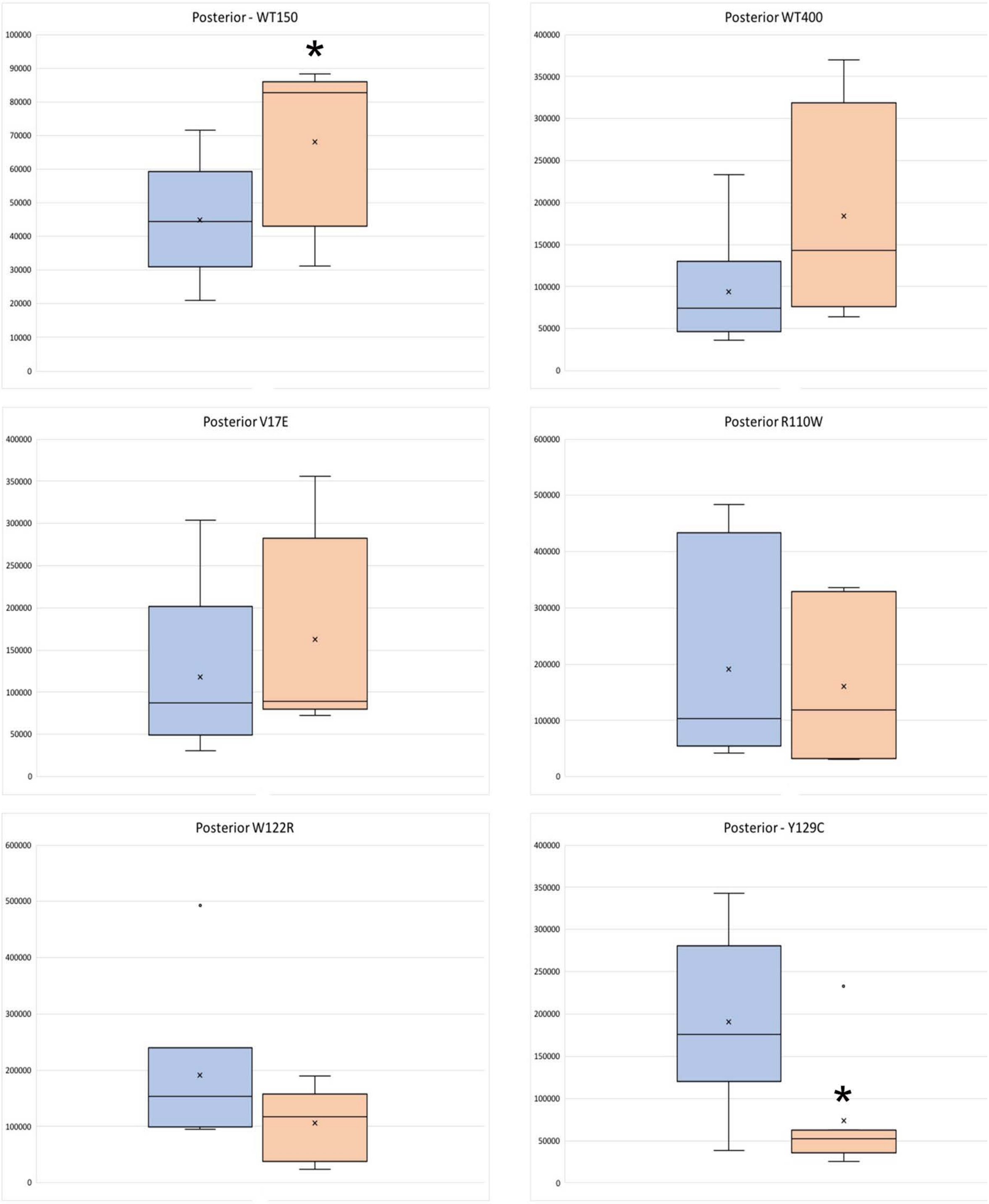

Disease Models & Mechanisms • Supplementary information

Supplemental Figure 1E

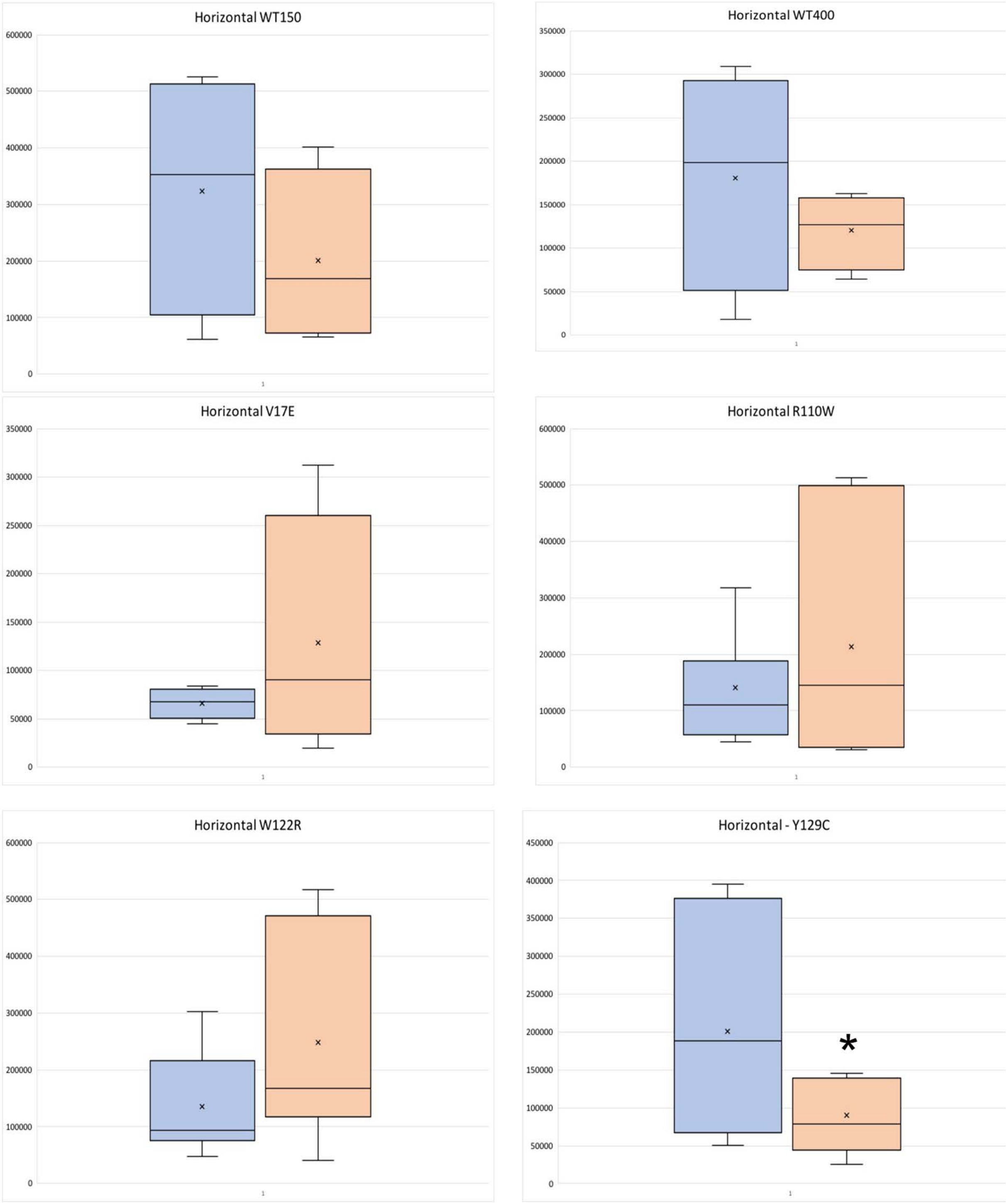

Disease Models & Mechanisms • Supplementary information

Figure S1: **Comparisons of sensory patch volumes (μm3) between manipulated (orange bar) and control (blue bar) sides of the same embryo.** **(A)** Saccule sensory patches. **(B)** Utricle sensory patches. **(C)** Anterior canal sensory patches. **(D)** Posterior canal sensory patches. **(E)** Horizontal canal sensory patches. Lines indicate medians, “x” indicates means, bars indicate standard errors. For most of the five end-organs measured there were no significant differences in volumes between the control, uninjected side and the *Six1* mutant mRNA-injected side of the embryo. There were trends for Six1WT- 150 to cause larger saccule and utricle sensory patches and smaller anterior canal and horizontal canal sensory patches, but these did not reach significance. However, Six1WT-150 did cause the posterior canal sensory patches to be significantly larger than control side. There were trends for Six1WT-400 to cause smaller saccule and anterior canal sensory patchesand larger posterior canal sensory patches, but these did not reach significance. However, Six1WT-400 did cause the utricular sensory patches to be significantly larger. The mutant proteins had no effect on saccule sensory patch volume. Utricle sensory patch volume trended smaller only with W122R. Anterior canal sensory patch volume was more variable with W122R and Y129C but these did not reach significance. Posterior canal sensory patch volume trended to slightly smaller with W122R and significantly smaller with Y129C. Horizontal canal sensory patch volume trended to more variable and larger with V17E, R110W and W122R without reaching significance, and was significantly smaller with Y129C. *, $p<0.05$.

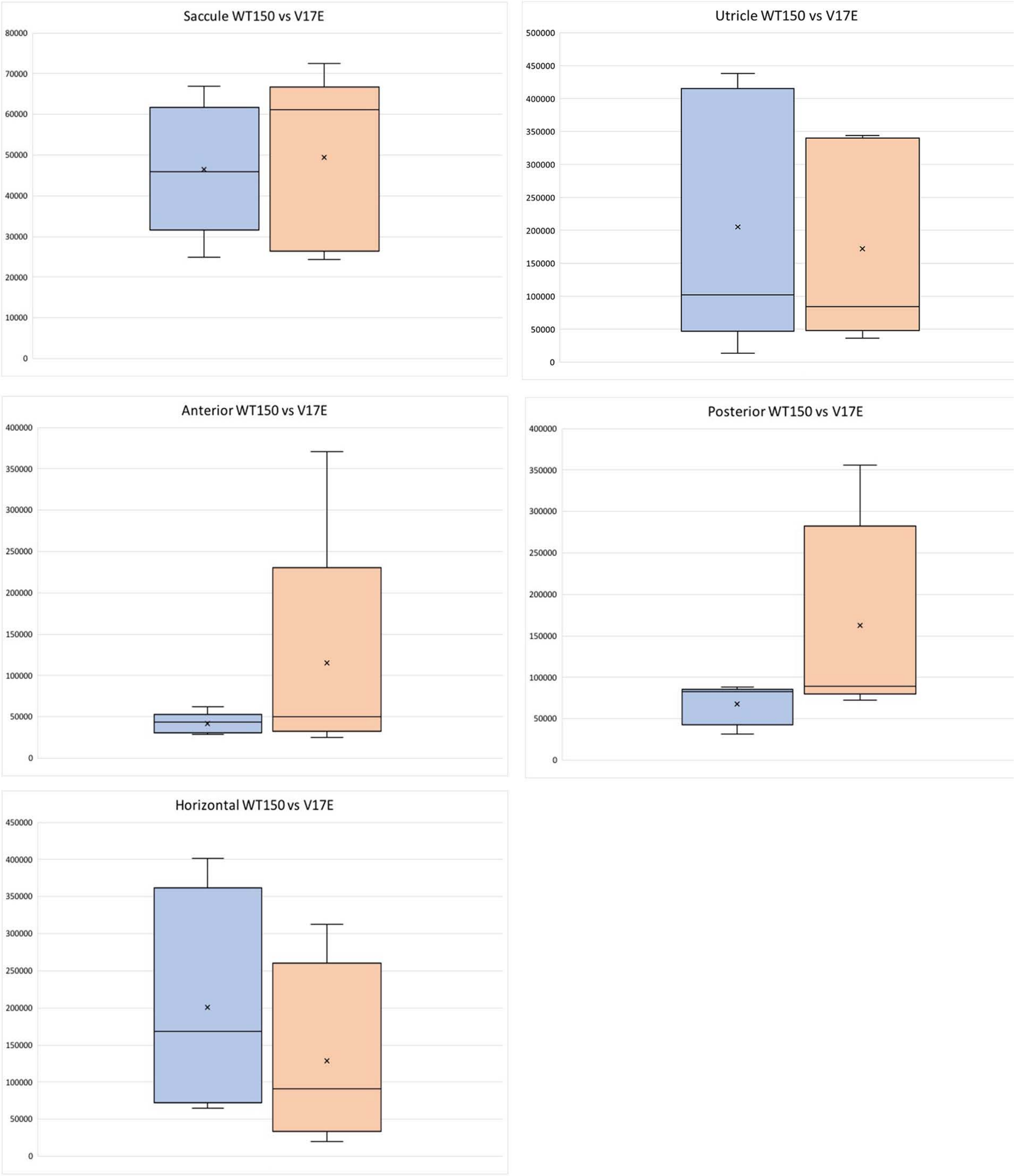
Saccule WT150 vs V17E
0
10000
20000
30000
40000
50000
60000
70000
80000
Utricle WT150 vs V17E
0
50000
100000
150000
200000
250000
300000
350000
400000
450000
500000
Anterior WT150 vs V17E
0
50000
100000
150000
200000
250000
300000
350000
400000
Posterior WT150 vs V17E
0
50000
100000
150000
200000
250000
300000
350000
400000
Horizontal WT150 vs V17E
0
50000
100000
150000
200000
250000
300000
350000
400000
450000

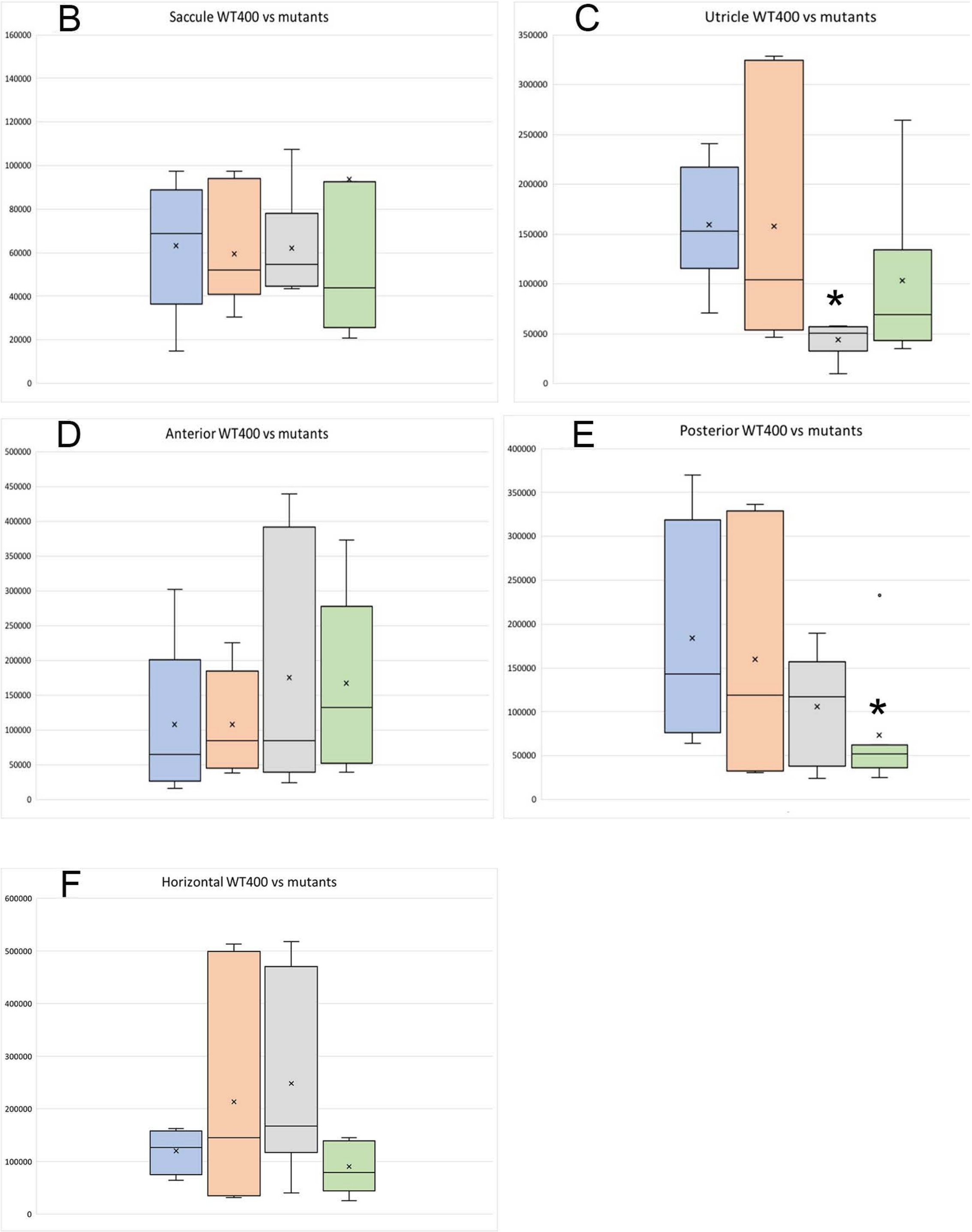

Disease Models & Mechanisms • Supplementary information

Figure S2: **Comparison of sensory patch volumes (µm3) volumes between Six1WT or Six1 mutant inner ears. (A)** Comparison between SixWT-150 (blue bars) and V17E (orange bars). Although there is a trend for the V17E anterior canal and posterior canal sensory patches to be larger than those of Six1WT-150, these differences did not reach significance (p>0.05, unpaired t-test). **(B)** Comparison between SixWT-400 (blue bar), R110W (orange bar), W122R (grey bar) or Y129C (green bar) saccule sensory patch volumes; no significant differences were detected (p>0.05, unpaired t-test). **(C)** Comparison between SixWT-400 (blue bar), R110W (orange bar), W122R (grey bar) or Y129C (green bar) utricle sensory patch volumes. R110W caused the largest variance and W122R and Y129C caused smaller volumes; only W122R reached significance (*, p<0.05). **(D)** Comparison between SixWT-400 (blue bar), R110W (orange bar), W122R (grey bar) or Y129C (green bar) anterior canal sensory patch volumes. No significant differences were detected. **(E)** Comparison between SixWT-400 (blue bar), R110W (orange bar), W122R (grey bar) or Y129C (green bar) posterior canal sensory patch volumes. R110W caused the largest variance and W122R and Y129C caused smaller volumes; only Y129C reached significance (*, p<0.05). **(F)** Comparison between SixWT-400 (blue bar), R110W (orange bar), W122R (grey bar) or Y129C (green bar) horizontal canal sensory patch volumes. R110W and W122R showed large variance, whereas Y129C caused a slight reduction. No significant differences were detected (p>0.05). Lines indicate medians, “x” indicates means, bars indicate standard errors.

Tables S1-S5. WT Six1 and mutant Six1 mRNA doses are identified at the top of each column.
For example, "V17E-150" indicates injection of 150 pg of V17E mRNA.
The data are otic tissue volumes (μm^3) on the control and injected sides of the same larva/tadpole.

Click here to Download Tables S1-S5

Disease Models & Mechanisms • Supplementary information
